# Compositional induced structural phase transitions in (1 − *x*)(K_0.5_Na_0.5_)NbO_3_–*x*(Ba_0.5_Sr_0.5_)TiO_3_ ferroelectric solid solutions

**DOI:** 10.1038/s41598-023-45713-z

**Published:** 2023-11-04

**Authors:** Satyaranjan Sahoo, Dhiren K. Pradhan, Shalini Kumari, Koyal Suman Samantaray, Charanjeet Singh, Anupam Mishra, Md. Mijanur Rahaman, Banarji Behera, Ashok Kumar, Reji Thomas, Philip D. Rack, Dillip K. Pradhan

**Affiliations:** 1 Department of Physics and Astronomy, National Institute of Technology Rourkela, Rourkela, Odisha 769008 India; 2 Department of Materials Science and Engineering, University of Tennessee, Knoxville, TN 37996 USA; 3 Department of Materials Science & Engineering, The Pennsylvania State University, University Park, PA 16802 USA; 4 Department of Physics, Indian Institute of Technology Indore, Indore, 453552 India; 5 CSIR-National Physical Laboratory, Dr. K. S. Krishnan Marg, New Delhi, 110012 India; 6 Academy of Scientific and Innovative Research (AcSIR), Ghaziabad, 201002 India; 7 Department of Materials Engineering, Indian Institute of Science, Bangalore, 560012 India; 8 Department of Materials Science and Engineering, University of Rajshahi, Rajshahi, 6205 Bangladesh; 9 School of Physics, Sambalpur University, Jyoti Vihar, Burla, 768019 India; 10 Division of Research and Development, Lovely Professional University, Jalandhar-Delhi G.T. Road, Phagwara, Punjab 144411 India; 11 School of Chemical Engineering and Physical Sciences, Lovely Professional University, Jalandhar-Delhi G.T. Road, Phagwara, Punjab 144411 India

**Keywords:** Ferroelectrics and multiferroics, Phase transitions and critical phenomena

## Abstract

Ferroelectric materials exhibiting switchable and spontaneous polarization have strong potential to be utilized in various novel electronic devices. Solid solutions of different perovskite structures induce the coexistence of various phases and enhance the physical functionalities around the phase coexistence region. The construction of phase diagrams is important as they describe the material properties, which are linked to the underpinning physics determining the system. Here we present the phase diagram of (K_0.5_Na_0.5_NbO_3_)–(Ba_0.5_Sr_0.5_TiO_3_) (KNN-BST) system as a function of composition and their associated physical properties. Lead-free (1 − *x*)KNN–*x*BST (0 ≤ *x* ≤ 0.3) solid solution ceramics were synthesized by conventional solid-state reaction technique. The X-ray diffraction and Raman spectroscopic studies indicate composition-dependent structural phase transitions from an orthorhombic phase for *x* = 0 to orthorhombic + tetragonal dual-phase (for 0.025 ≤ *x* ≤ 0.15), then a tetragonal + cubic dual-phase (*x* = 0.2) and finally a cubic single phase for *x* ≥ 0.25 at room temperature (RT). Among these, the orthorhombic + tetragonal dual-phase system shows an enhanced value of the dielectric constant at room temperature. The phase transition temperatures, orthorhombic to tetragonal (T_O-T_) and tetragonal to cubic (T_C_), decrease with the increase in BST concentrations. The ferroelectric studies show a decrease of both 2P_r_ and E_C_ values with a rise in BST concentration and *x* = 0.025 showed a maximum piezoelectric coefficient.

## Introduction

The piezoelectric effect realized in some dielectric materials is characterized by the conversion of mechanical energy into electrical energy and vice-versa. Ferroelectric materials are non-linear dielectrics and are also a sub-group of piezoelectric materials, which have spontaneous and electrically switchable polarization (P)^1,2^. Thus, at present, multifunctional ferroelectric materials are widely used in numerous electrical/electronic devices such as sensors, electrostrictive actuators, electro-mechanical transducers, multilayer ceramic capacitors, integrated non-volatile memory and high-density energy storage devices^1,2^. The widely used ferroelectric materials for device applications are lead-based material systems, viz., PbTiO_3_, Pb(Zr_*x*_Ti_1−*x*_)O_3_ (PZT), (1 − *x*)Pb(Mg_1/3_Nb_2/3_O_3_)–*x*PbTiO_3_ (PMN–*x*PT), Pb_1−*x*_La_*x*_(Zr_*y*_T_1−*y*_)_1−*x*/4_O_3_ (PLZT) due to their excellent di-, ferro-, and piezo-, electric properties^1–6^. Most of the Pb-based ferroelectric materials used in the industry contain more than 60 wt% of lead. Therefore, the high volatility lead-based ferroelectrics pose an environmental concern and are also detrimental to human health^4–6^. To create awareness of environmental safety, restrictions were imposed by Waste of Electrical and Electronic Equipment^5^ and Restriction of certain Hazardous Substances directives^4,5,7^ on the hazardous Pb-based material systems used in various electronic devices. These legislations limit the use of lead and have forced researchers across the globe to search for alternative lead-free ferroelectric systems. The potential lead-free ferroelectrics such as BaTiO_3_ (BT), (K_0.5_Bi_0.5_)TiO_3_ (KBT), (Na_0.5_Bi_0.5_)TiO_3_ (NBT), and (K_0.5_Na_0.5_)NbO_3_ (KNN) systems are being investigated by different research groups around the world^2–12^. Among the above-mentioned systems, KNN has received significant attention as an alternative lead-free ferroelectric system after the important work of Saito et al*.* in Li, Ta, and Sb co-substituted KNN ((K_0.44_Na_0.52_Li_0.04_)(Nb_0.84_Ta_0.10_Sb_0.06_)O_3_) textured ceramic revealed excellent piezoelectric properties (d_33_ as high as 416 pC/N)^11^. KNN is also a room-temperature ferroelectric material with good ferroelectric properties with a high remnant polarization (P_r_ = 33 μC/cm^2^), high Curie temperature (T_C_ ~ 420 °C), and moderate piezoelectric coefficient (d_33_ = 80 pC/N)^13^.

KNN, the solid solution of KNbO_3_ and NaNbO_3,_ crystallizes into the orthorhombic crystal structure with space group (SG) *Amm2* at RT for the morphotropic phase boundary (MPB) composition^14,15^. The KNN system undergoes a sequence of phase transitions as a function of temperature and is analogous to phase transitions in barium titanate, as reported from temperature-dependent X-ray and neutron diffraction data^15^. With decreasing temperature, it transforms from paraelectric cubic (SG: $Pm\overline{3 }m$) to ferroelectric tetragonal (SG: *P4mm*) with a ferroelectric transition temperature T_C_ ~ 415 °C, then from ferroelectric tetragonal (SG: *P4mm*) to ferroelectric orthorhombic (SG: *Amm2*) with a transition temperature T_O-T_ ~ 200 °C, after that ferroelectric orthorhombic (SG: *Amm2*) to ferroelectric rhombohedral (SG: *R3m*) with transition temperature (T_O-R_) ~  − 150 °C^14,15^. A few studies have also reported monoclinic structure (SG: *Pm*) for the KNN system^16–18^. The structural phase transition behavior in KNN has also been studied using various experimental techniques such as X-ray absorption fine structure^19^, transmission electron microscopy (TEM)^20^, nuclear magnetic resonance (NMR)^21^, electron paramagnetic resonance (EPR)^22^, Raman spectroscopic studies^23,24^. For the KNN system, the orthorhombic-tetragonal phase (T_O-T_) boundary is known as a polymorphic phase boundary (PPB), which is quite different from an MPB^14^. It is believed that the enhancement of the physical properties in the KNN system is due to the decrease in T_O-T_ approaching RT^25^. However, piezoelectric properties are also very sensitive to temperature fluctuations around a PPB. As a PPB strongly affects the physical properties of the KNN system, tailoring the transition temperature (i.e., T_O-T_) towards room temperature is one of the effective ways to enhance the physical properties for practical applications^25^. It has been established that the phase transitions in ferroelectric systems can be modified using external stimuli viz., chemical substitution, temperature, pressure, electric field, and also with swift heavy ions (SHI)^2,3,7,12^.

Solid solutions of KNN with various simple perovskite oxides, viz., Bi_0.5_K_0.5_TiO_3,_ Bi_0.5_Na_0.5_TiO_3_, Bi_0.5_Li_0.5_TiO_3_, BiAlO_3_, BiScO_3_, BaTiO_3_, SrTiO_3_, CaTiO_3_, LiNbO_3_, LiSbO_3_, LiTaO_3_ have been synthesized to improve the density as well as the piezoelectric properties for practical applications at room temperature and to understand the compositionally induced phase transitions^7,12–14,24–35^. For a brief summary of the various KNN-based solid solutions, readers are referred to Supplementary Table S1. Park et al*.* studied the compositional driven structural phase transition of (1 − *x*)(K_0.5_Na_0.5_)NbO_3_–*x*CaTiO_3_ ceramics and reported the highest d_33_ (~ 241 pC/N) in the orthorhombic and tetragonal phase coexistence region^28^. A structural phase transition from orthorhombic to tetragonal phase with the formation of an MPB has been reported in (1 − *x*)(K_0.5_Na_0.5_)NbO_3_–*x*Bi_0.5_K_0.5_TiO_3_ solid solutions^29^. The observed maximum d_33_ (251 pC/N) at *x* = 0.2 is due to the formation of an MPB and shifting of PPT near RT^29^. Liang et al*.* examined the microstructural and dielectric properties of (1 − *x*)(K_0.5_Na_0.5_)NbO_3_–*x*BiScO_3_ solid solutions and reported the relaxor nature of the ceramics^30^. The structural and electrical properties of (1 − *x*)(K_0.5_Na_0.5_)NbO_3_-*x*LiNbO_3_ ceramics were investigated by Liang et al*.* and they observed an enhanced piezoelectric constant (d_33_ = 200–235 pC/N) and electromechanical coefficient near the MPB region (0.05 < *x* < 0.07)^31^. The structural and dielectric properties of (1 − *x*)K_0.5_Na_0.5_NbO_3_–*x*SrTiO_3_ ceramics were studied by Kosec et al*.* and they reported the relaxor behavior in the composition range of *x* = 0.15 to 0.25^32^. Based on the Rietveld refinement of the XRD data, Sun et al*.* reported a compositional-driven structural phase transition in (1 − *x*)KNN–*x*BS ceramics with the formation of an MPB. In this MPB region, enhanced ferroelectric (P_r_ = 24.4 μC/cm^2^) and piezoelectric properties (d_33_ = 203 pC/N) are observed^33^. Park et al*.* studied the structural and electrical properties of 0.95(K_0.5_Na_0.5_)NbO_3_–0.05BaTiO_3_ ceramics and reported a high piezoelectric constant (d_33_ = 225 pC/N) in the MPB region^34^. A structural phase transition from orthorhombic to pseudo-cubic phase with the formation of an MPB was observed in (1 − *x*)(K_0.5_Na_0.5_)NbO_3_–*x*BiAlO_3_ ceramics. In the MPB region, a peak in the ferroelectric (P_r_ = 23.6 μC/cm^2^) and piezoelectric properties (d_33_ = 202 pC/N) were reported^35^.

In the present work, ferroelectric solid solutions of KNN with perovskite Ba_0.5_Sr_0.5_Ti0_3_ (BST) are considered. The Ba^2+^ cation radius (0.161 nm, CN = 12) is close to K^+^ (0.164 nm, CN = 12), and the cationic radius of Sr^2+^ (0.144 nm, CN = 12) is close to that of Na^+^ (0.139 nm, CN = 12)^36^. As in the case of KNN, the K and Na ratio is 1:1, so we have proposed a Ba:Sr ratio of 1:1. Ba_0.5_Sr_0.5_Ti0_3_ is in the paraelectric phase with cubic structure at RT^36^. Thus, a sequence of phase transitions from orthorhombic to cubic is expected as the BST concentration increases in the KNN-BST solid solutions. To the best of our knowledge, only one report is available on the (1 − *x*) (K_0.5_Na_0.5_NbO_3_)–*x*(Ba_0.5_Sr_0.5_TiO_3_) (*x* ≥ 10 mol%) solid solution, which was prepared and characterized by Du et al. They reported that the addition of BST induces the compositional-driven structural phase transition from orthorhombic to tetragonal, then to pseudo-cubic, and finally to cubic system^36^. Similarly, they also report the relaxor behavior in KNN-BST solid solutions^36^. An in-depth study on compositional-driven structural phase transitions (quantitatively) in KNN-BST solid solution as well as its co-relation with the physical properties, has not been clearly studied from the structural perspective. Here, we have extensively investigated the compositional-driven phase transition quantitatively by Rietveld refinement, which has been further corroborated with the Raman spectroscopy analysis. The physical properties and phase transition behaviors have also been interpreted based on the structural analysis. Finally, a phase diagram is presented over a wide range of compositions.

## Experimental

### Synthesis procedure

Polycrystalline (1 − *x*)KNN–*x*BST, (where *x* = 0.00, 0.025, 0.05, 0.10, 0.15, 0.20, 0.30) ceramics were prepared using the conventional solid-state reaction technique. Stoichiometric amounts of high purity K_2_CO_3_ (99% Alfa Aesar), Na_2_CO_3_ (99.5% Sigma Aldrich), Nb_2_O_5_ (99.99% Sigma Aldrich), BaCO_3_ (99.8% Alfa Aesar), SrCO_3_ (99.9% Sigma Aldrich), and TiO_2_ (99% Sigma Aldrich) were used as the precursors. Prior to weighing, the raw materials were dried at 220 °C for 4 h to remove the moisture intake. After weighing in stoichiometric ratios, the precursors were mixed and ground using an agate mortar and pestle, both dry and in an acetone medium, for about 4 h. The ground powders were then placed in an alumina crucible and calcined at 875 °C (optimized temperature) for 6 h in ambient conditions to realize the desired solid-state reaction. The calcined powder was again ground, mixed with 3 wt% polyvinyl alcohol (PVA), and then pressed into cylindrical disks of 10 mm diameter using a uniaxial hydraulic press. The green pellets were then sintered at 1175 °C for 3 h, which is the optimum sintering condition for the densification.

### Characterization techniques

Room temperature XRD measurements were performed on the powder samples using a Rigaku Smartlab X-ray diffractometer with Cu K_α1_ radiation (λ = 1.5405 Å). For XRD measurement, the sintered pellets were crushed into fine powder and then sieved. After that, the powders were heated at 500 °C to release the intergranular stress due to the grinding process. The data was collected in the two theta range 15° to 120° with a step size of 0.01 at a scan rate of 2°/min. The RT Raman spectra of the sintered pellets were measured using a micro Raman spectrometer (Invia, Renishaw, UK). Field emission scanning electron microscopy (FESEM) was used to study the surface microstructure of the sintered ceramics (FESEM, JOEL Inc., #IT800). For electrical characterization, both sides of the sintered samples were polished, painted with silver paste (to act as an electrode), and then dried at 200 °C to realize ohmic contacts. The dielectric properties over a wide frequency range (100 Hz to 1 MHz) were recorded using an LCR meter (HIOKI IM3570) in the temperature range RT-500 °C. For ferroelectric and piezoelectric measurements, the silver-electroded samples were poled in a silicone oil bath at an electric field of around 20 to 28 kV/cm, about 16 h at room temperature. We pole for longer than usual times because we poled the samples at room temperature, which is much lower than the ferroelectric T_C_. The ferroelectric hysteresis loop (P-E) measurements were performed at RT using a Radiant Ferroelectric Tester at 5 Hz. The piezoelectric coefficient d_33_ of the poled samples was measured using a Berlincourt piezometer (Piezotest PM-300).

## Results and discussion

### Structural studies: X-ray powder diffraction

Pure KNN undergoes successive phase transitions as a function of temperature^12,13^ and hence a similar type of structural phase transition is also expected for the ferroelectric solid solution of (1 − *x*)KNN–*x*BST. Thus, XRD and Raman analyses were carried out on the KNN-BST solid solutions as a function of BST content. Further, Rietveld refinements have been performed to fit XRD data for quantitative analysis of the structural phase transition with the addition of BST in KNN. The XRD patterns of KNN-BST samples are shown in Fig. S1. The XRD patterns exhibit the characteristic peaks corresponding to the perovskite phase without the presence of any secondary phases. This suggests that BST has diffused into the KNN lattice, forming the solid solution of the proposed compositions.

Scrutiny of splitting of the pseudocubic (pc) XRD reflections {h00}_pc,_ {hh0}_pc_, and {hhh}_pc_ is the best method for determining the presence of different crystallographic phases such as cubic, tetragonal, and orthorhombic. It has been reported that for the cubic symmetry, all the pseudocubic reflections are singlet, whereas the splitting of the {h00}_pc_ into doublets and singlet nature of the {hhh}_pc_ reflections are characteristic features of the tetragonal symmetry. Furthermore, the {hh0}_pc_ reflection is a doublet for the tetragonal phase, with the stronger peak occurring at the lower 2θ side. For the rhombohedral crystal structure, the reverse trend is observed, i.e., doublet nature of {hhh}_pc_ reflections with singlet nature of {h00}_pc_ reflections. On the other hand, the orthorhombic crystal structure shows the splitting of {h00}_pc,_ {hh0}_pc_ and {hhh}_pc_ reflections into doublets^6,37^.

**Figure 1:**
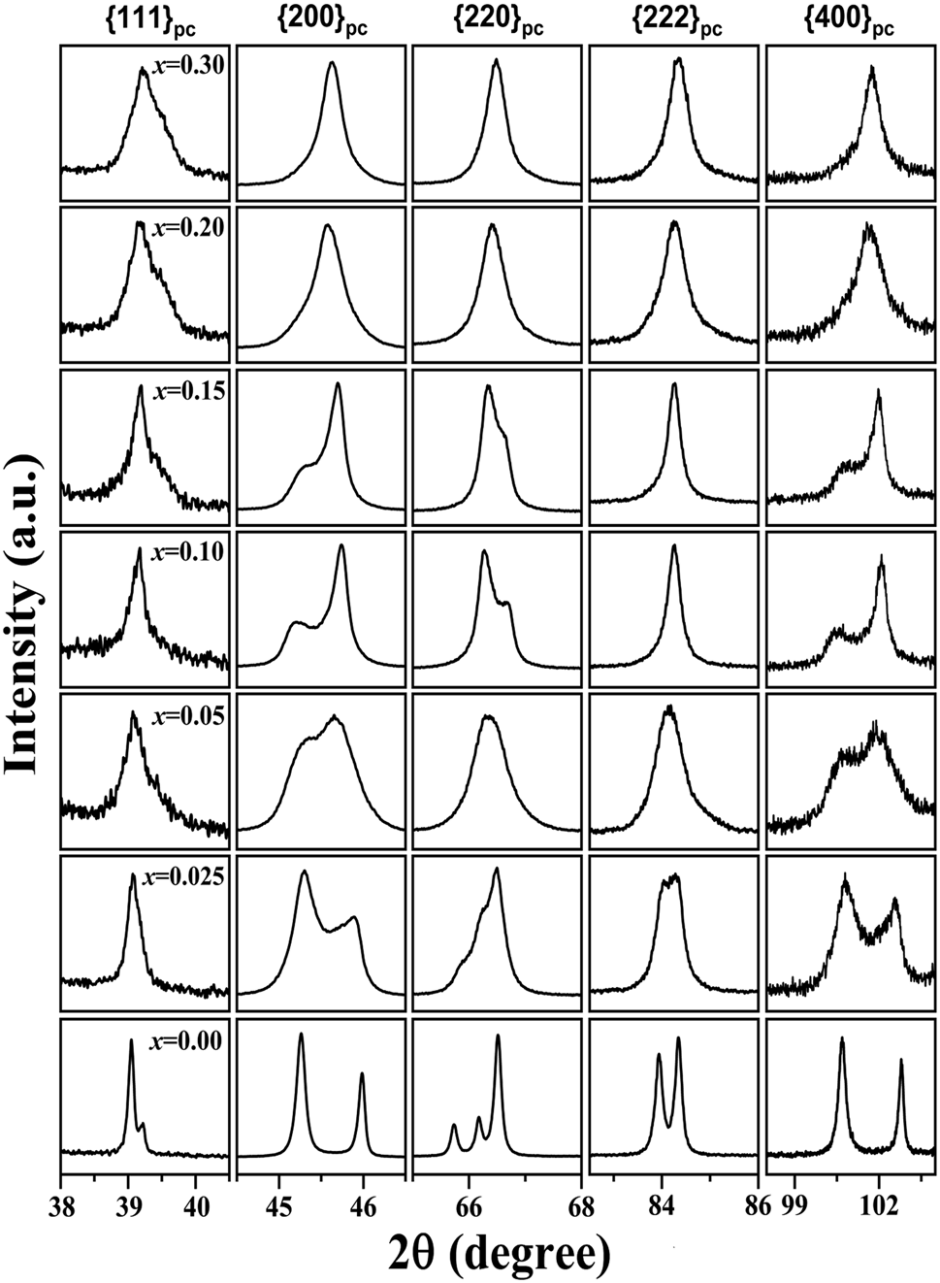
Evolution of XRD profiles of {111}_pc_, {200}_pc_, {220}_pc_, {222}_pc_, and {400}_pc_ pseudocubic reflections of (1 − *x*)KNN–*x*BST with 0 ≤ *x* ≤ 0.3 at RT.

Figure 1 represents the compositional evolution of the XRD profile for the pc reflections {111}_pc_, {200}_pc_, {220}_pc_, {222}_pc_, and {400}_pc_ of (1 − *x*)KNN–*x*BST ceramics in the composition range 0 ≤ *x* ≤ 0.3. In the XRD profiles, for the lower order reflections (lower 2θ), the splitting of the peaks is small for weak distortions of the lattice, whereas the splitting is more visible at higher order reflections. Hence, we have focused our attention on the {200}_pc_, {220}_pc_, {222}_pc_, and {400}_pc_ reflections.

For pure KNN (*x* = 0), the {200}_pc_ and {400}_pc_ reflections are doublets, and they split into (200)/(002) and (400)/(004) with a 2:1 intensity ratio. Further, the opposite trend in the peak splitting is observed in the {220}_pc_ reflection (compared to {200}_pc_ reflection). Concurrently, the {222}_pc_ reflection splits into a doublet. The splitting of {200}_pc_ and {400}_pc_ reflections in a 2:1 intensity ratio and the doublet nature of {222}_pc_ reflection clearly indicate the orthorhombic distortion of the unit cell^6,37^. Hence, pure KNN has orthorhombic crystal structure consistent with the literature at RT^14^. For *x* = 0.025, a similar trend in the XRD profile is observed. However, the gap between the {200}_pc_ reflections, i.e., (200)/(002) decreases, and the {222}_pc_ reflection broadens, suggesting the presence of an additional phase along with the orthorhombic phase. As the BST content increases, a distinct change in the profile shapes occurs for *x* = 0.05, where the splitting of {200}_pc_ and {400}_pc_ reflections are not clearly resolved, along with the broadening of these reflections. A close look at the {200}_pc_ and {400}_pc_ reflections suggests that (002) and (004) peaks emerge with enhanced intensity compared to the (200) and (400) peaks. Concurrently, the {220}_pc_ and {222}_pc_ peaks appear as a singlet. The opposite trend in the peak splitting of {200}_pc_ and {400}_pc_ reflections and the singlet nature of {222}_pc_ reflection may suggest that the orthorhombic distortion of KNN gradually decreases along with the appearance of another crystallographic structure (maybe tetragonal phase) with BST substitution. For *x* = 0.10 and 0.15, the intensity of the (002) and (004) peaks further increases (compared to the (200) and (400) peaks), and the {222}_pc_ reflection becomes a singlet. The splitting of {200}_pc_ and {400}_pc_ reflections into (200)/(002) and (400)/(004) with a 1:2 intensity ratio and singlet nature of the {222}_pc_ reflection indicate the tetragonal crystal structure^37^. With further increase in *x* (*x* ≥ 0.20), all the profiles become singlet, which suggests the cubic structure^6,37^.

**Figure 2:**
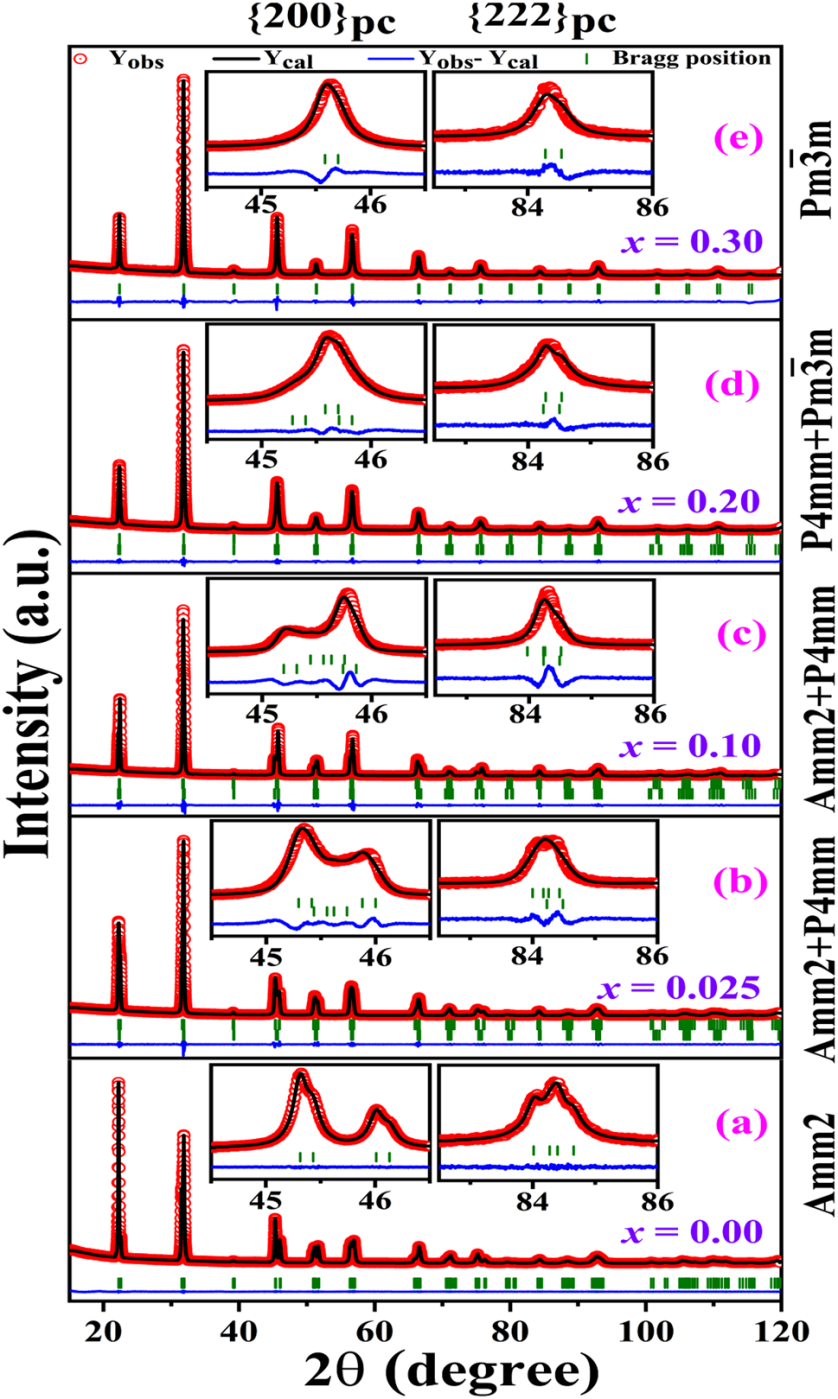
Rietveld refined X-ray powder diffraction patterns of (1 − *x*)KNN-*x*BST for **(a)** *x* = 0 using *Amm2* model **(b)** *x* = 0.025 and **(c)** *x* = 0.10 using *Amm2* + *P4mm* model **(d)** *x* = 0.20 using *P4mm*+$Pm\overline{3 }m$ model and **(e)** *x* = 0.30 using $Pm\overline{3 }m$ model. The inset shows a magnified view of the fitted {200}_pc_ and {222}_pc_ reflections.

Based on the visual observation of the XRD spectra, the noticeable change observed in the pseudo-cubic reflections with increasing *x* suggests a composition-induced structural phase transition from orthorhombic to tetragonal and subsequently to cubic structure. To determine the exact nature of this phase transition, the Rietveld refinement analysis of the XRD patterns was performed using the FULLPROF software^6,38^ which is shown in Fig. 2. Here, the starting models for the refinement were proposed from the visual inspections of the XRD spectra (as explained above). The pseudo-Voigt function was used to model the peak shape, whereas the background shape was refined using the linear interpolation technique for the refinement. Various refined parameters such as zero correction, scale factor, lattice parameters, Wyckoff positions, background, and half-width parameters (U, V, and W) were refined while keeping the occupancy constant during the refinement.

For *x* = 0, the XRD data was fitted using Rietveld refinement technique with single-phase *Amm2* crystal structure, and the result shows a good agreement between the experimental and the selected structural model (Fig. 2a). A magnified view of the fitted data of the higher order reflections {200}_pc_ and {222}_pc_ are shown in the insets of Fig. 2. Although from visual analysis of the doublet nature of the pseudocubic {200}_pc_ and {400}_pc_ reflections suggest an *Amm2* structure for *x* = 0.025 (Fig. 1), Rietveld analysis revealed a poor fit with the single phase *Amm2* structure. A dramatic improvement in the peak fit occurs by including the *P4mm* phase along with the *Amm2* phase (Fig. 2b). However, we have also fitted the XRD data for *x* = 0.025 by considering single phase *Amm2*, single phase *P4mm*, and combination of both *Amm2* and *P4mm* (*Amm2* + *P4mm*) phases. The best fit was observed for the (*Amm2* + *P4mm*) co-existence model. The Rietveld refined XRD patterns for single-phase *Amm2* and dual-phase (*Amm2* + *P4mm*) are shown in Supplementary Fig. S2. For *x* = 0.05, Rietveld refinement of the XRD patterns continued with *Amm2* + *P4mm* space groups, and a good fit was also observed for this dual phase. Although *x* = 0.10 and 0.15 look to be tetragonal, the Rietveld refinement using the single phase *P4mm* space group resulted in a poor fit. The best quality of fit was achieved using a combination of both tetragonal *P4mm* and orthorhombic *Amm2* phases. Figure 2c shows the representative Rietveld refinement pattern for *x* = 0.10. The magnified fitted plot of {200}_pc_ and {222}_pc_ reflections is also presented in the inset of Fig. 2c for better clarity. It was observed that the phase fraction of the tetragonal phase increases with increasing BST concentration in the phase co-existence region. The pseudo-cubic reflections for *x* = 0.20 appear to be cubic, so we have fitted that data using $Pm\overline{3 }m,$ which resulted in a very poor fit. Thus, the Rietveld refinement for *x* = 0.20 was carried out using different models such as (i) single phase $Pm\overline{3 }m$, (ii) single phase *P4mm*, and (iii) both $Pm\overline{3 }m$ and *P4mm* phases (*P4mm* + $Pm\overline{3 }m$)*.* The Rietveld refinement fit was improved when the tetragonal phase is included along with the cubic phase in the structural model. Thus the Rietveld refinement for *x* = 0.20 was carried out using dual phase $Pm\overline{3 }m$ and *P4mm* phases (*P4mm* + $Pm\overline{3 }m$), and the result shows a good agreement between the experimental and chosen theoretical model (Fig. 2d). For comparison we have also shown the fitted data using both $Pm\overline{3 }m$ and *P4mm*+$Pm\overline{3 }m$ phases in the Supplementary Fig. S3. Finally, Rietveld refinements of the XRD pattern for *x* = 0.30 with single phase cubic $Pm\overline{3 }m$ space group has been carried out, and a good fit has been observed (Fig. 2e). The refined structural parameters obtained for all the Rietveld refinements are given in Table S2 in the Supplementary Material.

Summarily, the Rietveld refinement on the XRD analysis reveals that (1-*x*)KNN-*x*BST ceramic exhibits the single phase *Amm2* crystal structure for *x* = 0, the coexistence of *Amm2* + *P4mm* structure for the composition range 0.025 ≤ *x* ≤ 0.15, the coexistence of *P4mm*+$Pm\overline{3 }m$ structure for *x* = 0.20 and single phase $Pm\overline{3 }m$ crystal structure for *x* = 0.30. We have also checked the presence of all elements and their respective valence states of *x* = 0.1 sample as representative of the KNN-BST system via high-resolution X-ray photoelectron spectroscopy (XPS). All the elements are found to be present, and their original valence states have not been changed significantly (Supplementary Material Fig. S4).

### Raman spectroscopic study

Raman spectroscopy is a nondestructive, effective probe to investigate the structural phase transition of ferroelectric materials because of its sensitivity to structural symmetry^39^. Raman scattering is also responsive to local heterogeneities associated with compositional and structural disorder. Thus, the composition-dependent Raman scattering spectra of the KNN-BST system have been investigated to realize the effects of BST concentration on local heterogeneities of KNN-BST ceramics. The reduced intensity, ${{I}}^{\text{r}}\left(\omega \right)$, corrected for the Bose–Einstein phonon population and observed Raman scattering intensity, *I*(ω) are related by the following equation^40,41^.

1 $${\text{I}}^{\text{r}}\left(\omega \right)\text{ } = \frac{{\text{I}}\text{(}\omega \text{)}}{\omega \text{[}{\text{n}}\left(\omega \right)\text{+1]}},$$

where, ${\text{n}}\left(\omega \right)\text{ = }\frac{1}{{\text{exp}}\left(\frac{\hbar \omega }{{\text{k}}_{\text{B}}{\text{T}}}\right)-1}$ stand for the Bose–Einstein population factor in which *k*_B_ and *ħ* denote Boltzmann and Dirac constants, respectively. All reduced Raman spectra in the frequency range of 25–100 cm^−1^ were fitted by admixture of a Lorentzian central peak (CP) and damped harmonic oscillators (DHOs) model to comprehend the effects of BST doping on KNN-BST ceramics^40,41^:

2 $$I^{r} \left( \omega \right){ = }\frac{{{2}A_{{{\text{CP}}}} }}{\pi }\frac{{\Gamma_{{{\text{CP}}}} }}{{{4}\omega^{{2}} { + } \Gamma_{{{\text{CP}}}}^{{2}} }} + \mathop \sum \limits_{{\text{i}}} \frac{{A_{{\text{i}}} \Gamma_{{\text{i}}} \omega_{{\text{i}}}^{{2}} }}{{\left( {\omega^{{2}} - \omega_{{\text{i}}}^{{2}} } \right)^{{2}} { + }\omega^{{2}} \Gamma_{{\text{i}}}^{{2}} }},$$

where *Γ*_CP_ and *A*_CP_ represent the full width at half maximum (FWHM) and intensity of the CP, respectively, which is associated with the relaxation process of precursor dynamics. $\omega$_i_,* Γ*_i_, and *A*_i_ represent frequency, damping constant, and intensity of the ith optical Raman active mode, respectively.

**Figure 3:**
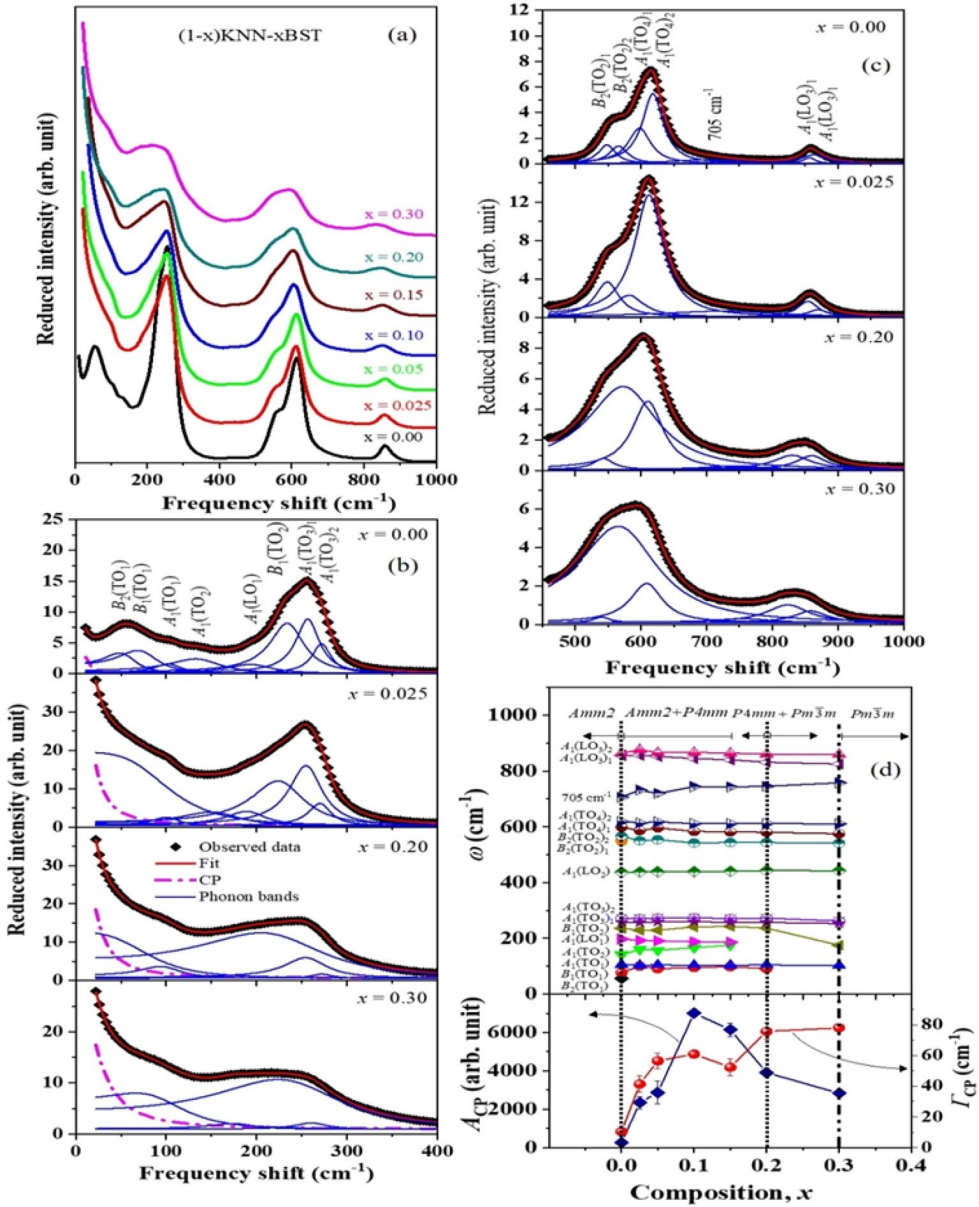
**(a)** Raman spectra of KNN-*x*BST ceramics as a function of composition. **(b, c)** The fitted Raman spectra using Eq. (2) at some selected composition. **(d)** The frequency shift (upper part) of Raman active modes, and the FWHM and intensity of the CP (lower part) of KNN-*x*BST ceramics as a function of composition.

The observed Raman scattering spectra measured at room temperature of the KNN-*x*BST ceramics as a function composition is shown in Fig. 3a. The Rietveld refinements of the XRD spectrum reveal that KNN belongs to the orthorhombic phase with *Amm2* symmetry. The temperature dependence of the dielectric properties also suggests that KNN belongs to the orthorhombic phase at room temperature. According to group theory analysis, the *Amm2* symmetry has 12 optical modes at the zone center, and the irreducible representations are 4*A*_1_ + *A*_2_ + 4*B*_1_ + 3*B*_2_^42,43^. In polycrystalline ceramic samples, it is difficult to assign the vibrational mode symmetries directly from the Raman scattering experiment; therefore, we follow the assignments of mode symmetry in [(K_0.56_Na_0.44_)(Nb_0.65_Ta_0.35_)O_3_, KNNT] single crystals^42^.

The composition-dependent Raman spectra of the KNN-*x*BST ceramics measured at room temperature are shown in Fig. 3a. The Raman spectrum in the frequency range of 25–1000 cm^−1^ of the KNN consist of mainly *B*_2_(TO_1_) (~ 58 cm^−1^),* B*_1_(TO_1_) (~ 79 cm^−1^), *A*_1_(TO_1_) (~ 106 cm^−1^), *A*_1_(TO_2_) (~ 141 cm^−1^), *A*_1_(LO_1_) (~ 198 cm^−1^), *B*_1_(TO_2_) (~ 236 cm^−1^), *A*_1_(TO_3_) (~ 260 cm^−1^), *A*_1_(LO_2_) (~ 438 cm^−1^), *B*_*2*_(TO_2_) (~ 550 cm^−1^), *A*_1_(TO_4_) (~ 619 cm^−1^), and *A*_1_(LO_3_) (~ 856 cm^−1^) as shown in Fig. 3a–c. The broad weak mode at about 705 cm^−1^ (Fig. 3a–c) may be due to the mismatch of ionic radii at crystallographically equivalent sites induced lattice disorder^44^. Note that *A*_1_(TO_3_), *B*_2_(TO_2_), *A*_1_(TO_4_), and *A*_1_(LO_3_) each modes splits into two modes denote as *A*_1_(TO_3_)_1_ (~ 257 cm^−1^) and *A*_1_(TO_3_)_2_ (~ 272 cm^−1^), *B*_2_(TO_2_)_1_ (~ 550 cm^−1^) and *B*_2_(TO_2_)_2_ (~ 569 cm^−1^), *A*_1_(TO_4_)_1_ (~ 599 cm^−1^) and *A*_1_(TO_4_)_2_ (~ 619 cm^−1^), *A*_1_(LO_3_)_1_ (~ 856 cm^−1^) and *A*_1_(LO_3_)_2_ (~ 884 cm^−1^), respectively. The Raman mode splitting may be due to the different local order regions in KNN ceramics^45^. The observed Raman modes of the KNN ceramics correspond to the orthorhombic phase^42,43^, which is supported by XRD and dielectric results. It is found that the *B*_2_(TO_1_) mode completely disappears, and *B*_2_(TO_2_)_1_ and *B*_2_(TO_2_)_2_ modes merge at *x* = 0.025 as shown in Fig. 3a,d. The complete disappearance of the *B*_2_(TO_1_) mode and merging of the *B*_2_(TO_2_) mode denote the structural change of the KNN-BST ceramics. It is important to note that an over-damped phonon mode appears near 99 cm^−1^ at *x* = 0.025 (Fig. 3b). The over-damped phonon may correspond to the *E*(TO_1_) mode of the tetragonal phase of the KNN-BST ceramics^6^. Thus, the disappearance of the *B*_2_(TO_1_) mode and the appearance of an over-damped *E*(TO_1_) mode indicates the structural change of the KNN-BST from an orthorhombic to a tetragonal phase. This is consistent with the Rietveld refinement of the XRD spectra of KNN-BST (0.025 ≤ *x* ≤ 0.15), which revealed the coexistence of the orthorhombic (*Amm2*) and tetragonal (*P4mm*) phases. The existence of the *B*_2_(TO_2_) may indicate the phase coexistence of orthorhombic (*Amm2*) and tetragonal (*P4mm*) phases in KNN-BST (0.025 ≤ *x* ≤ 0.15) ceramics. Further increasing the BST composition, both *A*_1_(TO_2_) cm^−1^ and *A*_1_(LO_1_) modes become closer to each other and eventually vanish at *x* ≥ 0.20 (Fig. 3b,d). The vanishing of these Raman modes above *x* = 0.20 is a clear indication of structural transformation from tetragonal to cubic phase. The phase transition and the coexistence of tetragonal and cubic $(Pm\overline{3 }m$) phases i.e., *P4mm* + $Pm\overline{3 }m$ phase at *x* = 0.20 are confirmed by Rietveld refinement of the XRD spectra of KNN-*x*BST (*x* = 0.20) ceramics. It is worth noting that the overdamped *E*(TO_1_) mode corresponds to the tetragonal phase and still exists at *x* = 0.20. The presence of *E*(TO_1_) mode may imply the coexistence of tetragonal and cubic phases at *x* = 0.20 by Raman scattering as well. Note that the overdamped *E*(TO_1_) mode completely disappeared at *x* = 0.30, indicating the structural transformation from mixed *P4mm*+$Pm\overline{3 }m$ phases to a pure cubic $(Pm\overline{3 }m)$ phase. The first-order Raman mode is not allowed in cubic $Pm\overline{3 }m$ symmetry according to Raman selection rules. However, the intense first-order Raman modes persist in the cubic phase at *x* ≥ 0.30. The presence of first-order Raman modes in the cubic phase KNN–*x*BST (*x* = 0.30) denotes the breaking of symmetry caused by the local polar clusters i.e., polar nano regions PNRs^40,41^.

The Raman spectra of the KNN–xBST can also be explained using the vibrational modes of isolated cations and coordination polyhedrons. In this case, the vibrations stem from the internal modes of NbO_6_/TiO_6_ octahedrons and the translational modes of K^+^/Na^+^/Ba^2+^/Sr^2+^ cations. The vibrations of NbO_6_/TiO_6_ octahedrons consist of *A*_1g_(ν_1_) + *E*_g_(ν_2_) + 2F_1u_(ν_3,_ ν_4_) + F_2g_ (ν_5_) + F_2u_(ν_6_), in which *A*_1g_(ν_1_), *E*_g_(ν_2_), and F_1u_(ν_3_) modes are stretching and the rest are bending modes^42,43^. The Raman modes in the low-frequency range lower than 200 cm^−1^ can be assigned to the translational modes of the K^+^/Na^+^/Ba^2+^/Sr^2+^ cations and rotation of the octahedron, while other internal vibrational modes of the octahedron appear in the high-frequency range of 200–900 cm^−1^. In the low-frequency region, the ν_6_ mode associated with NbO_6_/TiO_6_ octahedron may also appear, and the Raman ν_6_ mode corresponds to peaks near 141 cm^−1^^43^. The modes at about 58 cm^−1^ and 79 cm^−1^ are associated with the translational modes of K^+^/Na^+^/Ba^2+^/Sr^2+^ cations, whereas the mode near 198 cm^−1^ is related to K^+^/Na^+^/Ba^2+^/Sr^2+^ cations versus NbO_6_/TiO_6_ octahedron^43^. The mode at around 106 cm^−1^ is related to the rotational mode of the NbO_6_/TiO_6_ octahedron^42^. The modes near 236 cm^−1^ and 260 cm^−1^ were attributed to the ν_5_ mode. The modes at about 438 cm^−1^, 550 cm^−1^, 619 cm^−1^, and 705 cm^−1^ are identified as ν_4_, ν_2_, ν_1_, and ν_3_ modes of the NbO_6_/TiO_6_ octahedron, respectively^43^. The coupled ν_1_ + ν_5_ mode is commonly treated as the peak at 856 cm^−1^^43^. It is found that the intense ν_1_ (619 cm^−1^) and ν_5_ (260 cm^−1^) modes become weak upon increasing the BST composition (Fig. 3a). The intense ν_1_ and ν_5_ modes indicate the near-perfect NbO_6_/TiO_6_ octahedron of pure KNN (*x* = 0) belongs to *Amm2* symmetry^43^. It is important to note that ν_1_ and ν_5_ (260 cm^−1^) modes of the KNN split into two with the addition of BST. The splitting of the modes is likely due to the substitution of Nb ions with Ti ions, which leads to a distortion of the crystal structure of the KNN and breaks the symmetry of NbO_6_/TiO_6_ octahedron^42^. Also, note that the frequency of these modes shifts to lower frequency in a slightly scattered way with increasing the BST compositions (Supplementary Fig. S5). The slightly scattered values may be due to the existence of mixed phases. This result suggests that the substitution of Nb ions with Ti ions may weaken the binding strength of the octahedron, which is caused by increasing the distance between Nb/Ti cations and its coordinated oxygen due to their lattice mismatch.

The composition dependence of the CP, which is related to the relaxation process of dynamics PNRs, has been investigated to comprehend the nature of phase transition and the effects of BST composition on local heterogeneities in KNN–*x*BST ceramics. The presence of the CP is a common feature of either a crystal or a ceramic undergoing the order–disorder phase transition, while the soft mode phenomena denotes a displacive phase transition of ferroelectric materials. The low-frequency *B*_2_(TO_1_) mode near 58 cm^−1^ is found in the orthorhombic phase of pure KNN ceramics, while the overdamped *E*(TO_1_) mode near 91 cm^−1^ is observed in the mixed (orthorhombic + tetragonal) phases of the KNN–*x*BST (0.025 ≤ *x* ≤ 0.15) in this study. It is difficult to comment on the soft-mode nature of the low-frequency *B*_2_(TO_1_) and *E*(TO_1_) modes without temperature-dependent Raman scattering results. Thus, the presence of noticeable CP may indicate the order–disorder nature of the ferroelectric phase transition of KNN–*x*BST ceramics^40,41,46^. In the paraelectric cubic phase of ferroelectric materials, the dynamic PNRs start to appear at the so-called Burns temperature (*T*_B_)^47^. However, dynamic PNRs turn into static PNRs at an intermediate temperature (T^*^)^40,41^. In ferroelectric phases, these static PNRs develop into randomly oriented nano-domain states and transform into macro-domain states due to the freezing of local polarization^48,49^. As can be seen in the lower part of Fig. 3d, the *Γ*_CP_, which is related to the relaxation process of precursor dynamics^40,41,46^ increases at *x* = 0.025, is almost constant in the region; 0.025 ≤ *x* ≤ 0.15, and then increases when *x* increases to 0.30. It is expected that the correlation among nano-domain states may be strengthened in the same phase with increasing the BST concentration, owing to the increase of the number density and/or size of randomly oriented nano-domain states resulting in a decrease in the value of the *Γ*_CP_^6,50^. Note that the value of *Γ*_CP_ increases with the BST composition except in the range of 0.025 ≤ *x* ≤ 0.15 composition. The increase of the *Γ*_CP_ denotes the structural phase transition of KNN–*x*BST. It is significant that the value of *Γ*_CP_ is almost constant in the range of 0.025 ≤ *x* ≤ 0.15, where KNN–*x*BST belongs to (orthorhombic + tetragonal) phases. These results suggest that the correlation among nano-domain states may be broken and/or weaken resulting in the almost constant fluctuations of domain wall motion due to the coexistence of phases^6^.

### Microstructural studies

**Figure 4:**
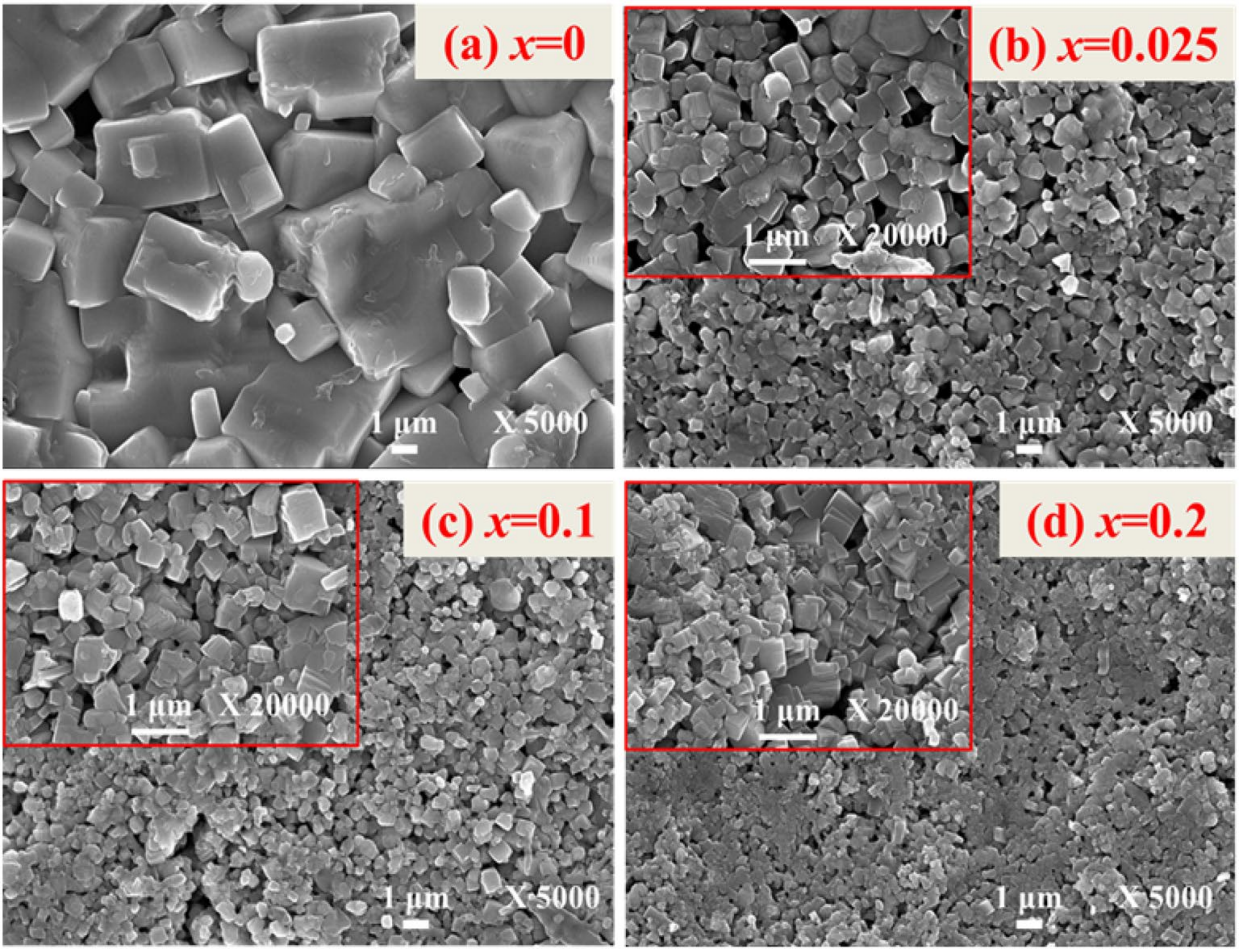
FESEM micrographs of the (1 − *x*)KNN–*x*BST ceramics for (**a**) *x* = 0, (**b**) *x* = 0.025, (**c**) *x* = 0.10, (**d**) *x* = 0.20.

The surface morphology of the ceramics was studied with FESEM. Figure 4 shows the selected FESEM micrographs of the (1 − *x*)KNN–*x*BST ceramics for *x* = 0, 0.025, 0.10, and 0.20 respectively (other compositions are shown in the Supplementary Material, Fig. S6). The FESEM image for KNN (*x* = 0) shows that due to abnormal grain growth, the microstructure consists of grains of different sizes because grain growth is different for different grains^25^. The well-defined grains for *x* = 0 suggest that the grain growth process is almost complete during the sintering process. However, the presence of few scattered pores cannot be avoided. The grain size decreases with an increase in BST concentration indicating that abnormal grain growth is reduced with BST^25^. The addition of BST results in a compact microstructure and uniform distribution of grains. The uniform fine-grained microstructure is suitable for higher mechanical strength and high electro-mechanical coefficient^51^. The average grain size is calculated using ImageJ software and it is found to be 2.52 μm (for *x* = 0). With increasing BST concentration the grain size reduces and for the higher BST compositions, the average grain size is calculated to be in the range 0.38–0.17 μm. See the summary of grain sizes in Table S3 in the Supplementary Section. We have measured the density of the ceramics by Archimedes’ principle. The density of the ceramics varies from 94 to 96% of the theoretical density.

### Dielectric studies

**Figure 5:**
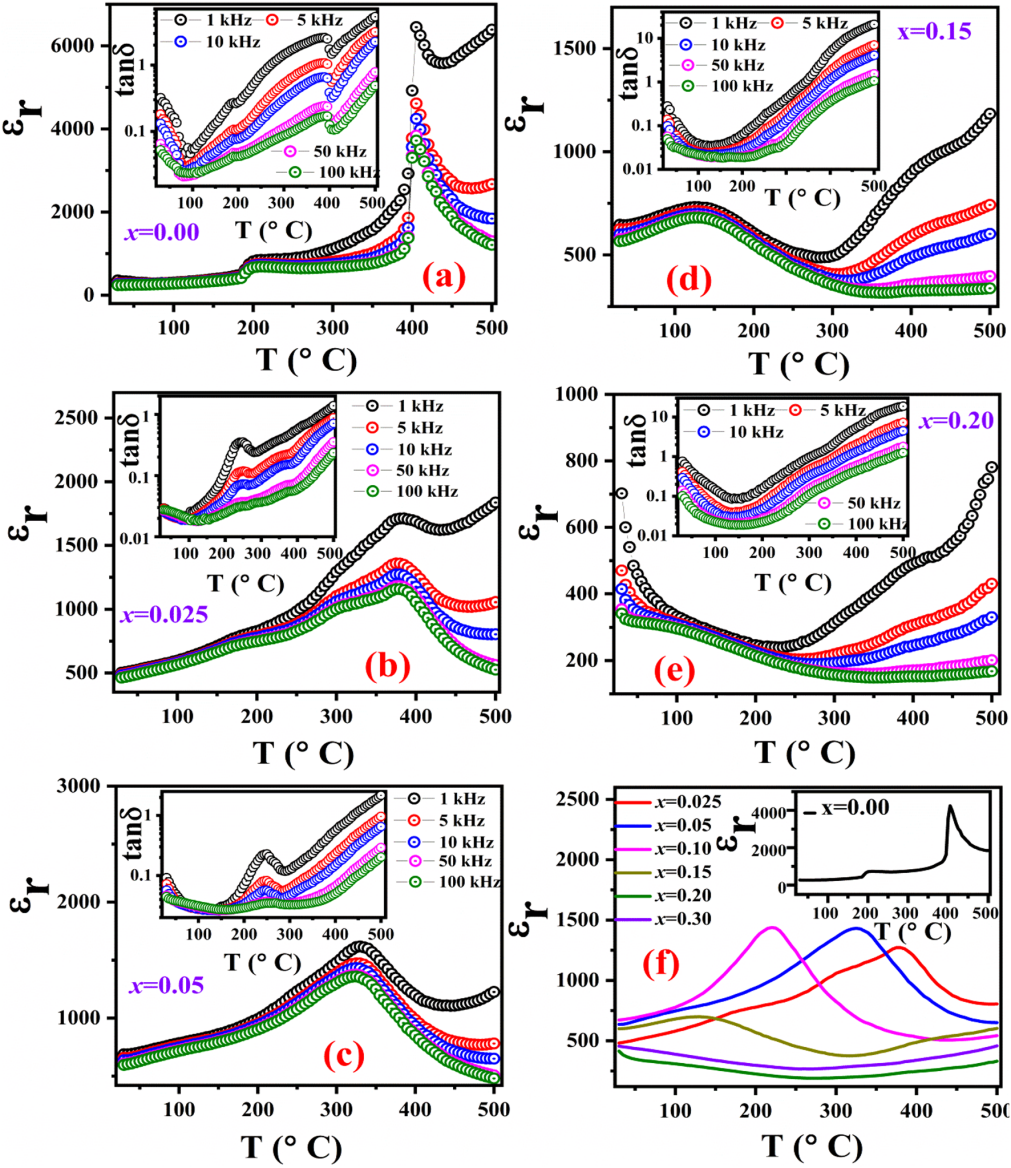
Variation of ε_r_ and tanδ as a function of temperature at selected frequencies of (1 − *x*)KNN–*x*BST ceramics for (**a**) *x* = 0, (**b**) *x* = 0.025, (**c**) *x* = 0.05, (**d**) *x* = 0.15, (**e**) *x* = 0.20 and (**f**) comparision of dielectric constant with temperature for all compositions at 10 kHz.

The variation of dielectric constant (ε_r_) and loss tangent (tanδ) as a function of temperature (T) at different frequencies were investigated to study the ferroelectric phase transition behavior in (1 − *x*)KNN–*x*BST solid-solutions (Fig. 5). The temperature dependence of the dielectric constant and loss tangent for* x* = 0, 0.025, 0.05, 0.15 and 0.20 at selected frequencies of 1 kHz, 5 kHz, 10 kHz, 50 kHz, and 100 kHz are shown in Fig. 5a–e (other compositions are shown in the Supplementary section, Fig. S7). The dielectric constant decreases with increasing frequency regardless of composition and temperature, which is a characteristic feature of polar dielectric materials^5^.

For pure KNN (*x* = 0), the ε_r_ versus temperature plot shows an increased value of ε_r_ with an increase in temperature, along with the appearance of two distinct anomalies in the measured temperature range. The first small anomaly around 210 °C corresponds to an orthorhombic to tetragonal (T_O-T_) phase transition, while the sharp peak around 405 °C relates to the ferroelectric tetragonal to paraelectric cubic phase transition (T_C_)^13,14^. The temperature-dependent dielectric loss also shows anomalies around the same temperatures, which is shown in the inset of Fig. 5a. The appearance of a peak in the temperature-dependent dielectric constant (ε_r_) and loss tangent (tanδ) confirms the ferroelectric phase transitions. For the ceramics with *x* = 0.025 and 0.05, the two anomalies corresponding to T_O-T_ and T_C_ are observed similar to *x* = 0 (Fig. 5b,c). However, both the T_O-T_ and T_C_ shift to the lower temperature and the tetragonal-cubic transition peaks broaden. Compared to 210 °C for pure KNN, the T_O-T_ is reduced to 170 °C for *x* = 0.025 and 85 °C for *x* = 0.05. Furthermore, the T_C_ values shift to 375 °C and 325 °C for *x* = 0.025 and *x* = 0.05, respectively; suggesting the possibility to lower the T_O-T_ to room temperature at higher BST concentration.

As the BST content increases further, the ε_r_ versus T plot for *x* = 0.10 and 0.15 do not show any phase transition corresponding to T_O-T_ and the only peak corresponding to tetragonal-cubic transition is observed (Fig. S7a, Fig. 5d). This suggests that, T_O-T_ shifts below the room temperature and the T_C_ value is found to be 220 °C and 130 °C for *x* = 0.10 and 0.15 respectively. However, the ε_r_ versus T plot for *x* = 0.20 does not show a clear transition corresponding to T_C_, which suggests that the material is in the paraelectric phase at RT. For *x* = 0.20, though Rietveld refinement of the XRD data shows the existence of both tetragonal and cubic phases (*P4mm* + $Pm\overline{3 }m$), however, the tetragonal phase fraction is quite low. Therefore, the dominant contribution from the cubic symmetry is responsible for the paraelectric phase at RT. For higher concentrations of BST i.e. for *x* = 0.3, the phase transition corresponding to T_C_ is also not visible (Fig. S7b), which may suggest that T_C_ decreases and shifts below the room temperature, which is beyond the investigated temperature range in the present study. The disappearance of the paraelectric-ferroelectric phase transition peak in the ε_r_ versus T plot suggests the cubic structure of *x* = 0.3 at room temperature which agrees with the XRD data.

For a clear observation of the effect of BST substitution on KNN ceramic, the temperature dependence of ε_r_ for the ceramics with 0 ≤ *x* ≤ 0.3 is plotted at a constant frequency of 10 kHz, which is shown in Fig. 5f where the inset is a plot of ε_r_ versus T for *x* = 0. Therefore, it can be concluded that, both T_O-T_ and T_C_ decrease systematically with the increase in BST concentration in KNN.

To confirm the nature of phase transition (normal/relaxor type), we have fitted the temperature-dependent dielectric permittivity data in the paraelectric region using modified Curie Wiess law. The modified Curie–Weiss equation^2,6^ can be expressed as

3 $$\frac{1}{{{\upvarepsilon }_{r} }} - \frac{1}{{{\upvarepsilon }_{m} }} = \frac{{\left( {T - T_{C} } \right)^{{\upgamma }} }}{C},$$

where ε_m_ = maximum value of ε_r_ at T_C_, C = modified Curie Weiss constant, and γ = degree of diffuseness. The value of γ can be found from the slope of the ln(1/ε_r_ − 1/ε_m_) versus ln(T − T_m_) plot and it varies from 1 to 2. The degree of diffuseness for normal ferroelectric materials is found to be 1, while it is 2 for very diffuse/relaxor type ferroelectric^2,6^.

The degree of diffuseness upon BST substitution in the present case has been extracted from the graphs of ln(1/ε_r_ − 1/ε_m_) versus ln(T − T_m_) at 10 kHz for the (1 − *x*)KNN–*x*BST ceramics (0 ≤ *x* ≤ 0.15) and are plotted in the paraelectric region as shown in Supplementary Fig. S8. From Fig. S8, the linear fit suggests that the modified Curie–Weiss law is satisfied. For *x* = 0, the value of γ is found to be 1.14, and the γ value increases with an increase in BST concentration (for example, γ = 1.77 for *x* = 0.15). The increase in γ value with ‘*x*’ represents the escalation in the diffuse phase transition, which suggests the relaxor-like behavior of the (1 − *x*)KNN–*x*BST solid solution^52^.

### Ferroelectric and piezoelectric properties

Ferroelectric properties of the (1 − *x*)KNN–*x*BST ceramic solid solutions were studied through the polarization versus electric field hysteresis loop (P–E loop) measurements at room temperature. The electrical poling close to the coercive field has been done prior to the P–E hysteresis measurements. The reason behind this pre-poling process is to orient the dipoles to obtain a net polarization in the material. It has been already reported that due to such electrical poling, the octahedra become more stable as it reduces the local structural heterogeneity and promotes long-range ferroelectric ordering. This, in fact, results in well-saturated ferroelectric hysteresis loops as can be seen elsewhere^2,8^.

**Figure 6:**
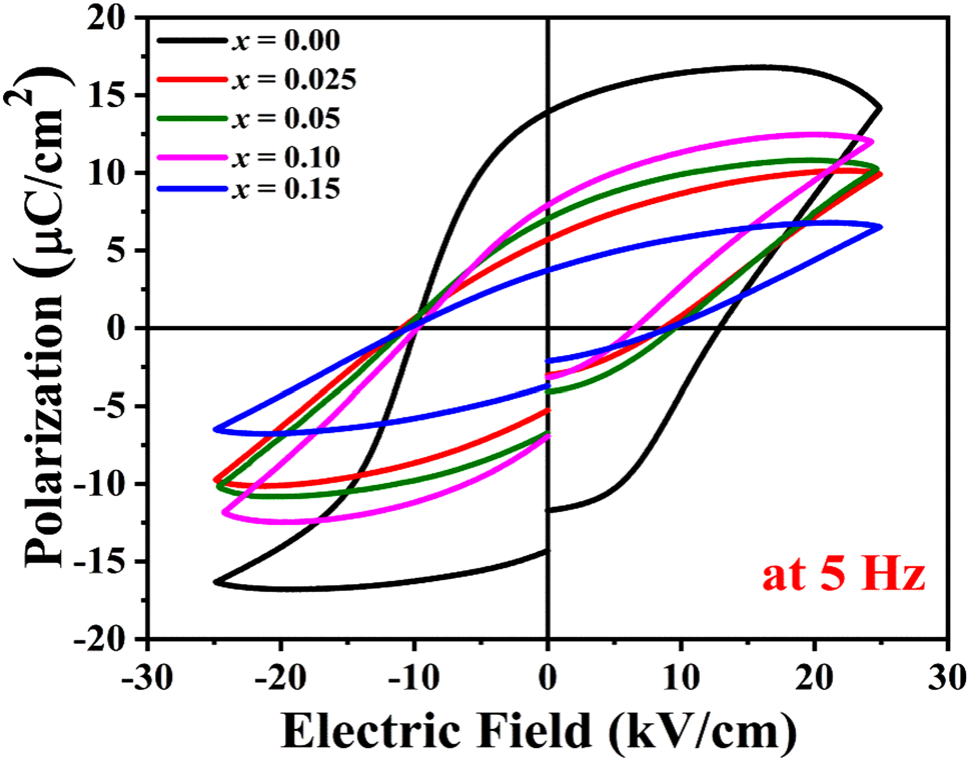
Ferroelectric hysteresis loop of poled samples of (1 − *x*)KNN–*x*BST ceramics at RT.

Figure 6 shows the RT *P–E* hysteresis loop of poled KNN-BST ceramics (0 ≤ *x* ≤ 0.15) at a frequency of 5 Hz. The *P–E* loop of pure KNN (*x* = 0) shows well-defined and non-linear behavior which suggests the ferroelectric nature. The partial unsaturated shape of the upper portion of pure KNN is due to the leakage through the large capacitive area with lossy-like behavior. However, with an increase in BST concentration, the area of the hysteresis loop decreases systematically. The PE loop for KNN (pure) showed higher 2P_r_ and E_C_ values compared to BST modified samples. By comparing the *P–E* hysteresis loops in different crystallographic phases for the KNN system, it has been reported that the remnant polarization (2P_r_) and coercive field (E_C_) are higher in the orthorhombic phase compared to the other crystallographic phases similar to the report of Shirane et al.^53^. For higher BST concentration i.e. for *x* = 0.2 and 0.3, we did not observe any saturated loops because of the paraelectric nature of the ceramics at room temperature. The ferroelectric parameters such as remnant polarization (2P_r_) and coercive field (E_C_) were calculated using the formula ${2P}_{r}^{0}= {P}_{r}^{+0}-{P}_{r}^{-0}$ and ${2E}_{C}^{0}= {E}_{C}^{+0}-{E}_{C}^{-0}$ and their variation with compositions along with the correlation with other physical properties will be discussed below.

**Figure 7:**
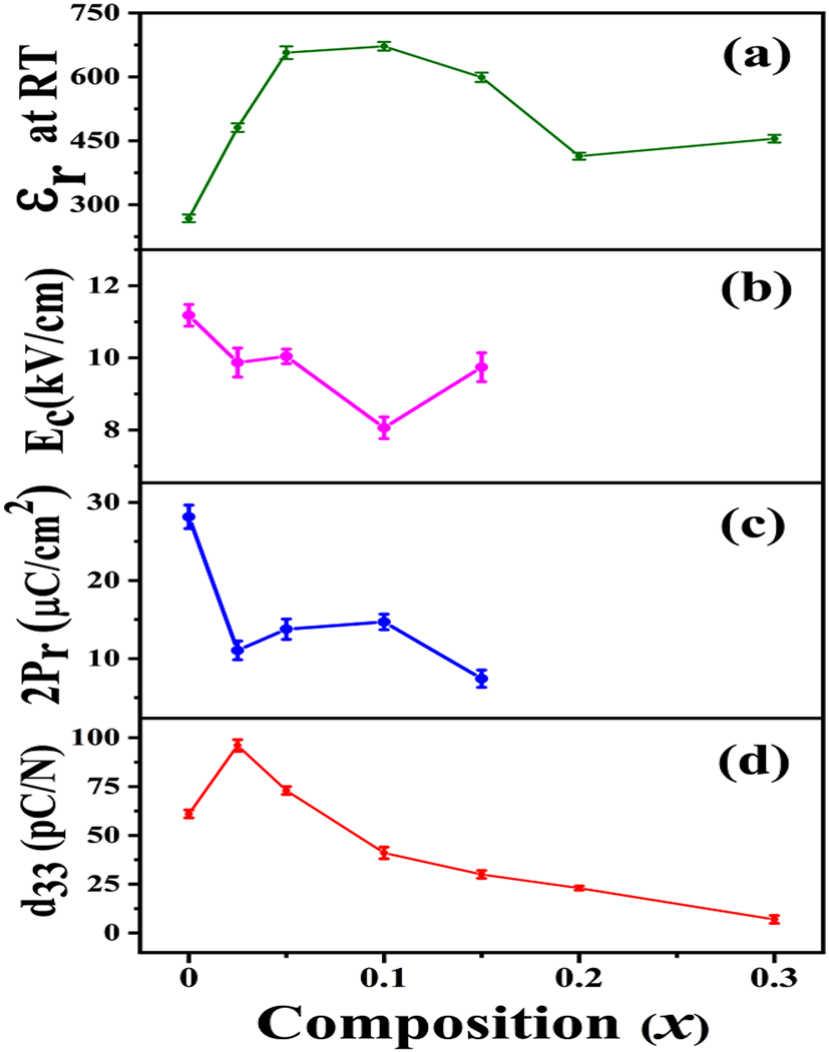
Variation of **(a)** dielectric constant, **(b)** coercieve field, **(c)** remnant polarization, and **(d)** piezoelectric coefficient with* x* for (1 − *x*)KNN–*x*BST ceramics at room temperature.

In order to study the effect of BST addition on the physical properties of (1 − *x*)KNN–*x*BST solid solutions, piezoelectric coefficient (d_33_), ferroelectric parameters (2P_r_ and E_C_) and dielectric constant (ε_r_) at room temperature were compared for different BST concentrations. Figure 7 shows the variation of dielectric constant, coercive field, remnant polarization and piezoelectric coefficient with *x* for the (1 − *x*)KNN–*x*BST ceramics with 0 ≤ *x* ≤ 0.3. For pure KNN (*x* = 0), the dielectric constant at RT is found to be 268. With increasing BST concentration, the dielectric constant, ε_r_ increases initially and reaches a maximum (ε_r_ = 672) for *x* = 0.1. After that, the ε_r_ versus *x* graph shows a gradual decrease in ε_r_ value up to *x* = 0.20 and then saturates. On the other hand, the ferroelectric properties (2P_r_ and E_C_) decrease with an increase in BST content. For pure KNN, relatively high 2P_r_ (28.15 μC/cm^2^) and high E_C_ (11.18 kV/cm) values are observed which may be due to the orthorhombic phase of KNN. With increasing BST concentration the E_C_ value decreases, is a minimum for *x* = 0.1 (E_C_ = 8.06 kV/cm) and subsequently it slightly increases. Similarly, the remnant polarization decreases with increasing *x*, and for *x* ≥ 0.05 it becomes nearly constant. Thus, with the addition of BST, the ferroelectric parameters decrease. However, the piezoelectric coefficient (d_33_) increases; for pure KNN, the d_33_ is found to be 61 pC/N. As the BST content increases, d_33_ increases sharply to 96 pC/N for *x* = 0.025, becomes maximum and after that, it decreases slowly with further increase in *x*.

The piezoelectric coefficient d_33_ for perovskite-based ferroelectric materials can be represented by the formula^54,55^

4 $$d_{33} = 2Q_{11} {\upvarepsilon }_{0} {\upvarepsilon }_{33} P_{3} ,$$

where P_3_ = polarization along the polar axis and approximately equals to P_r_ in this case, ε_0_ = dielectric permittivity in free space, ε_33_ = ε_r_ is the dielectric permittivity, and Q_11_ = electrostrictive coefficient which varies between 0.05 and 0.1 m^4^ C^−2^^55^. In the present case, with increasing BST concentration the ε_r_ increases and at the same time, 2P_r_ decreases (for the low value of *x*) due to the decrease in phase transition temperature. Since d_33_ is directly proportional to ε_r_, the increased value of ε_r_ leads to enhancement of d_33_. At the same time due to the direct dependence of d_33_ on P_r_, because P_r_ decreases linearly with *x*, d_33_ turns over and decreases for* x* > 0.025. Thus, the observed enhancement of d_33_ for *x* = 0.025 as expected is due to the simultaneous contribution from both increased ε_r_ and decreased value of 2P_r_. So the d_33_ value is a competition between these two parameters. A similar type of variations has also been observed in BT-modified KNN System^13^.

**Figure 8:**
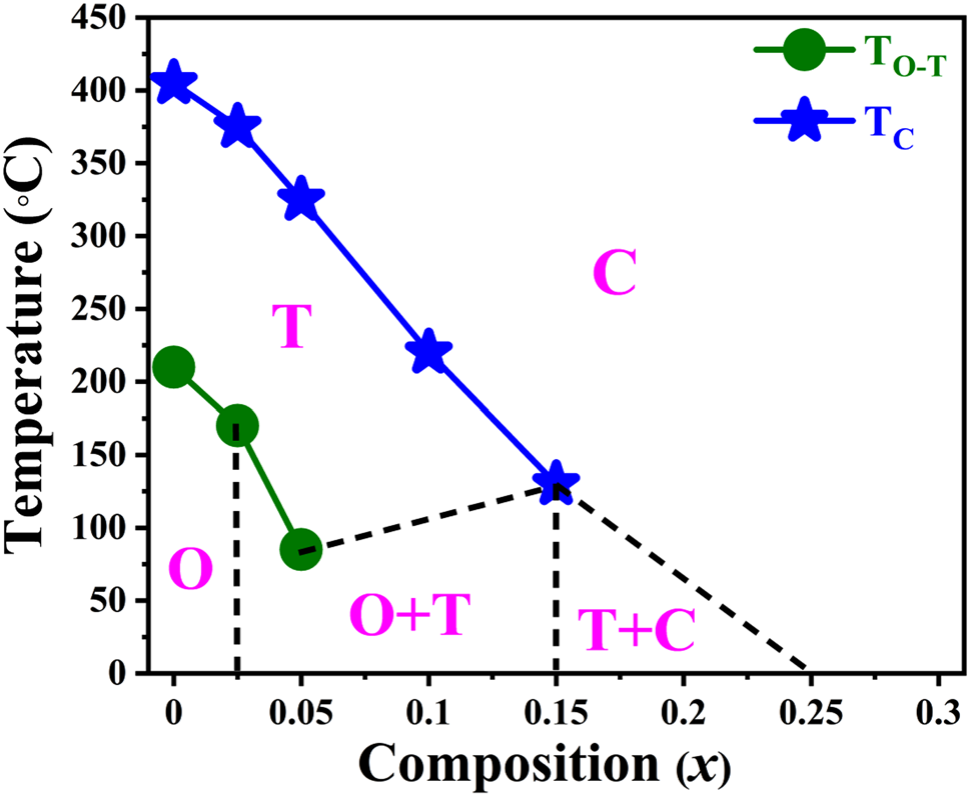
Phase diagram of (1 − *x*)KNN–*x*BST ceramics for 0 ≤ *x* ≤ 0.3. The labels O, T, and C denote orthorhombic, tetragonal and cubic phases respectively.

The detailed study correlating the structural phase transition behavior is presented in the phase diagram of (1 − *x*)KNN–*x*BST solid solutions as shown in Fig. 8. This phase diagram has been drawn based on the room temperature X-ray diffraction, Raman spectroscopy studies and the temperature-dependent dielectric properties discussed before. Pure KNN crystallizes in the orthorhombic structure (SG: *Amm2*) at RT. The phase diagram consists of a paraelectric cubic phase for the high temperature at a lower concentration of BST and at RT for a higher concentration of BST in (1 − *x*)KNN–*x*BST solid solutions.

With increasing BST concentration, KNN transforms from orthorhombic to phase coexistence region (i.e., orthorhombic + tetragonal), then to (tetragonal + cubic) phases and finally to cubic phase for *x* ≥ 0.25 (based on the XRD and Raman data). The phase coexistence region of the orthorhombic + tetragonal phase (0.025 ≤ *x* ≤ 0.15) forms a pentagon region above the pure orthorhombic phase. On the other hand, the coexistence region tetragonal + cubic forms a triangle below the cubic phase. In the coexistence region of the orthorhombic + tetragonal phase, the phase formation of the tetragonal phase increases with increasing BST composition and transforms to the tetragonal + cubic coexistence phase at *x* = 0.15. Finally, it transforms to a cubic phase at *x* ≥ 0.25. With increasing temperature, the orthorhombic phase of KNN transforms to the tetragonal phase and finally to the cubic paraelectric phase. However, the orthorhombic + tetragonal phase coexistence region transforms to the tetragonal phase and finally to the cubic phase. Similarly, the phase coexistence region of the tetragonal + cubic phase transforms to a cubic phase with increasing temperature.

## Conclusions

In summary, the effect of BST concentration on the structural, morphological, dielectric, ferroelectric and piezoelectric properties of (1 − *x*)KNN–*x*BST ceramics has been investigated over a wide range of compositions (0 ≤ *x* ≤ 0.3). The XRD and Raman spectroscopic studies suggest a compositional-driven structural phase transition from the orthorhombic (*Amm2*) phase to the orthorhombic + tetragonal (*Amm2* + *P4mm*) phase, then to the tetragonal + cubic (*P4mm* + $Pm\overline{3 }m$) phase and finally to cubic ($Pm\overline{3 }m$) phase with increase in BST concentration at room temperature. For pure KNN, the temperature-dependent dielectric properties show two phase transitions i.e., orthorhombic to tetragonal (T_O-T_) phase transition and tetragonal to cubic phase transition (T_C_). Both the phase transition temperatures decrease with increasing BST concentration and for *x* ≥ 0.1, T_O-T_ is expected to be below the RT. The dielectric constant at RT increases with BST concentration up to *x* = 0.1 and after that, it decreases. The ferroelectric hysteresis loop shows ferroelectric behavior up to *x* = 0.15 and paraelectric behavior for *x* = 0.25 and 0.30. The observed decrease in 2P_r_ and E_C_ values with an increase in BST concentration can be attributed to the centrosymmetric paraelectric phase of BST. A maximum piezoelectric coefficient (d_33_ = 96 pC/N) was observed for *x* = 0.025. A phase diagram has been presented based on the temperature-dependent dielectric, RT XRD, and Raman data.

### Supplementary Information

Supplementary Information.

## Data Availability

The datasets used and/or analysed during the current study available from the corresponding author on reasonable request.
