# Supplementary material for: Compositional induced structural phase transitions in (1 − x)(K0.5Na0.5)NbO3–x(Ba0.5Sr0.5)TiO3 ferroelectric solid solutions
Source: Sci Rep. 2023 Nov 4;13:19096. doi: 10.1038/s41598-023-45713-z (PMC10625606; doi:10.1038/s41598-023-45713-z)
Supplement: Supplementary file 1 — Supplementary Information. [file 41598_2023_45713_MOESM1_ESM.pdf]

Compositional Induced Structural Phase Transitions in (1-x)($K_{0.5}Na_{0.5}$)NbO_3-x($Ba_{0.5}Sr_{0.5}$)TiO_3 Ferroelectric Solid Solutions

Satyaranjan Sahoo[1], Dhiren K. Pradhan[2], Shalini Kumari[3], Koyal Suman Samantaray[4], Charanjeet Singh[5,6], Anupam Mishra[7], Md. Mijanur Rahaman[8], Banarji Behera[9], Ashok Kumar[5,6], Reji Thomas[10,11], Philip D. Rack[2], and Dillip K. Pradhan[1*]

[1]Department of Physics and Astronomy, National Institute of Technology Rourkela, Rourkela, Odisha 769008, India
[2]Department of Materials Science and Engineering, University of Tennessee, Knoxville, Tennessee 37996, USA
[3]Department of Materials Science & Engineering, The Pennsylvania State University, University Park, Pennsylvania 16802, USA
[4]Department of Physics, Indian Institute of Technology Indore, Indore, 453552, India
[5]CSIR-National Physical Laboratory, Dr. K. S. Krishnan Marg, New Delhi 110012, India
[6]Academy of Scientific and Innovative Research (AcSIR), Ghaziabad 201002, India
[7]Department of Materials Engineering, Indian Institute of Science, Bangalore, 560012, India
[8]Department of Materials Science and Engineering, University of Rajshahi, Rajshahi 6205, Bangladesh
[9]School of Physics, Sambalpur University, Jyoti Vihar, Burla,768019, India
[10]Division of Research and Development, Lovely Professional University, Jalandhar-Delhi G.T. Road, Phagwara, Punjab 144411, India
[11]School of Chemical Engineering and Physical Sciences, Lovely Professional University, Jalandhar-Delhi G.T. Road, Phagwara, Punjab 144411, India

Figure Captions (Supplementary Material)

Figure S1. Room temperature XRD patterns of (1-*x*)KNN-*x*BST ceramics ($0 \leq x \leq 0.3$).

Figure S2. Rietveld refined x-ray powder diffraction pattern of 0.975KNN-0.025BST- using (a) *Amm2*, and (b) *Amm2+P4mm* model. The inset shows magnified view of the fitted $\{200\}_{pc}$ and $\{222\}_{pc}$ reflections.

Figure S3. Rietveld fitted x-ray powder diffraction pattern of 0.8KNN-0.2BST using (a) $Pm\overline{3}m$, and (b) *P4mm*+ $Pm\overline{3}m$ model. The inset shows magnified view of the fitted $\{200\}_{pc}$ and $\{222\}_{pc}$ reflections.

Figure S4. XPS spectra of each element in 0.9KNN-0.1BST solid solution (a) K 2p, (b) Na 1s, (c) Nb 3d, (d) Ba 3d, (e) Sr 3d, (f) Ti 2p, (g) O 1s and (h) C 1s.

Figure S5. The frequency shift of the $A_1(TO_3)$ and $A_1(TO_4)$ modes as a function of composition.

Figure S6. FESEM micrographs of the (1-*x*)KNN-*x*BST ceramics for (a) $x = 0.05$, (b) $x = 0.15$, and (c) $x = 0.30$.

Figure S7. Variation of dielectric constant and dielectric loss as a function of temperature at selected frequencies of (1-*x*)KNN-*x*BST ceramics for (a) $x = 0.10$, (b) $x = 0.30$.

Figure S8. Modified Curie Wiess law plot ($\ln(1/\varepsilon_r - 1/\varepsilon_m)$ versus $\ln(T-T_m)$) of the (1-*x*)KNN-*x*BST ceramics for (a) $x = 0$, (b) $x = 0.025$, (c) $x = 0.05$, (d) $x = 0.1$, and (e) $x = 0.15$

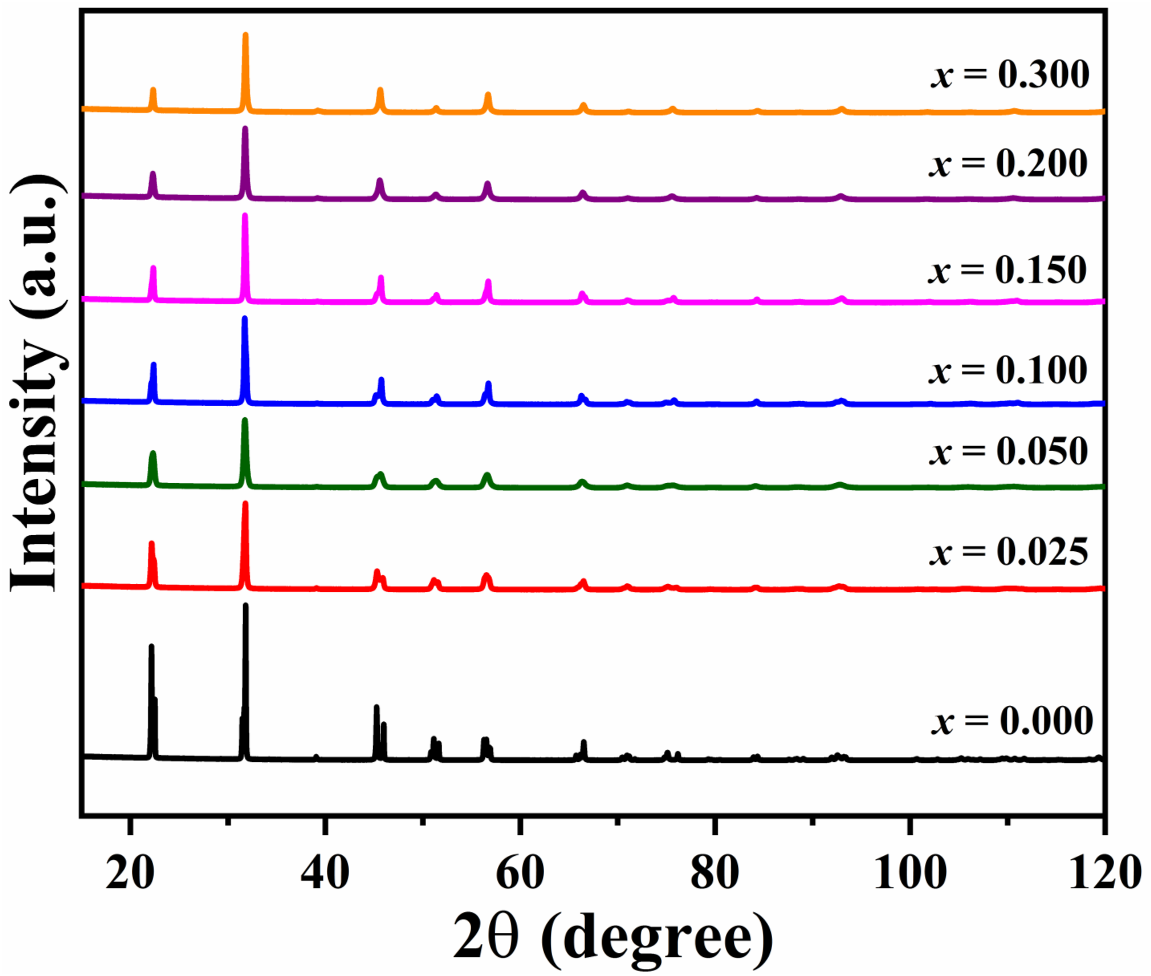

Figure S1. Room temperature XRD patterns of (1-*x*)KNN-*x*BST ceramics ($0 \leq x \leq 0.3$).

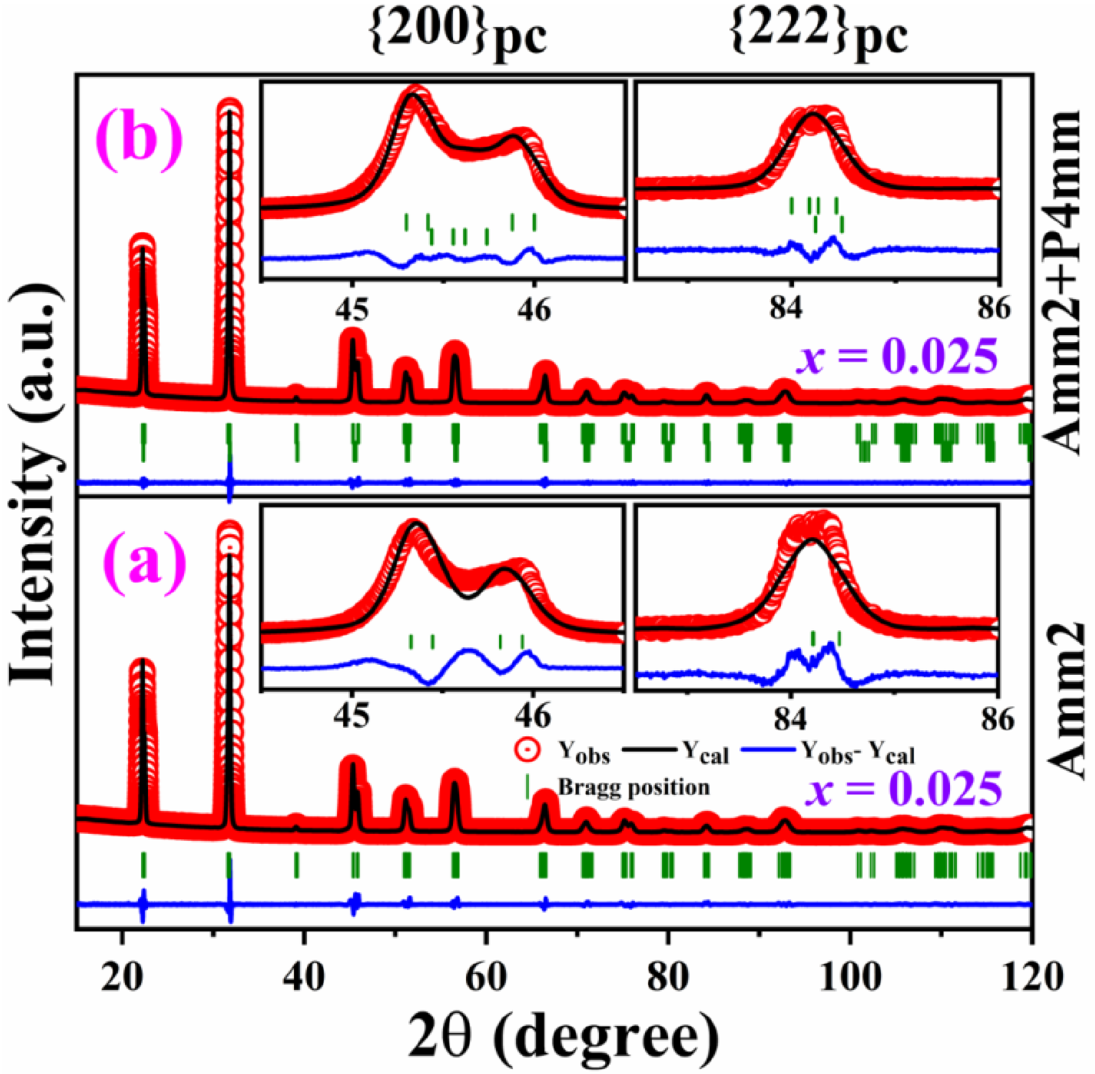

Figure S2. Rietveld refined x-ray powder diffraction pattern of 0.975KNN-0.025BST using **(a)** *Amm2*, and **(b)** *Amm2+P4mm* model. The inset shows magnified view of the fitted $\{200\}_{pc}$ and $\{222\}_{pc}$ reflections.

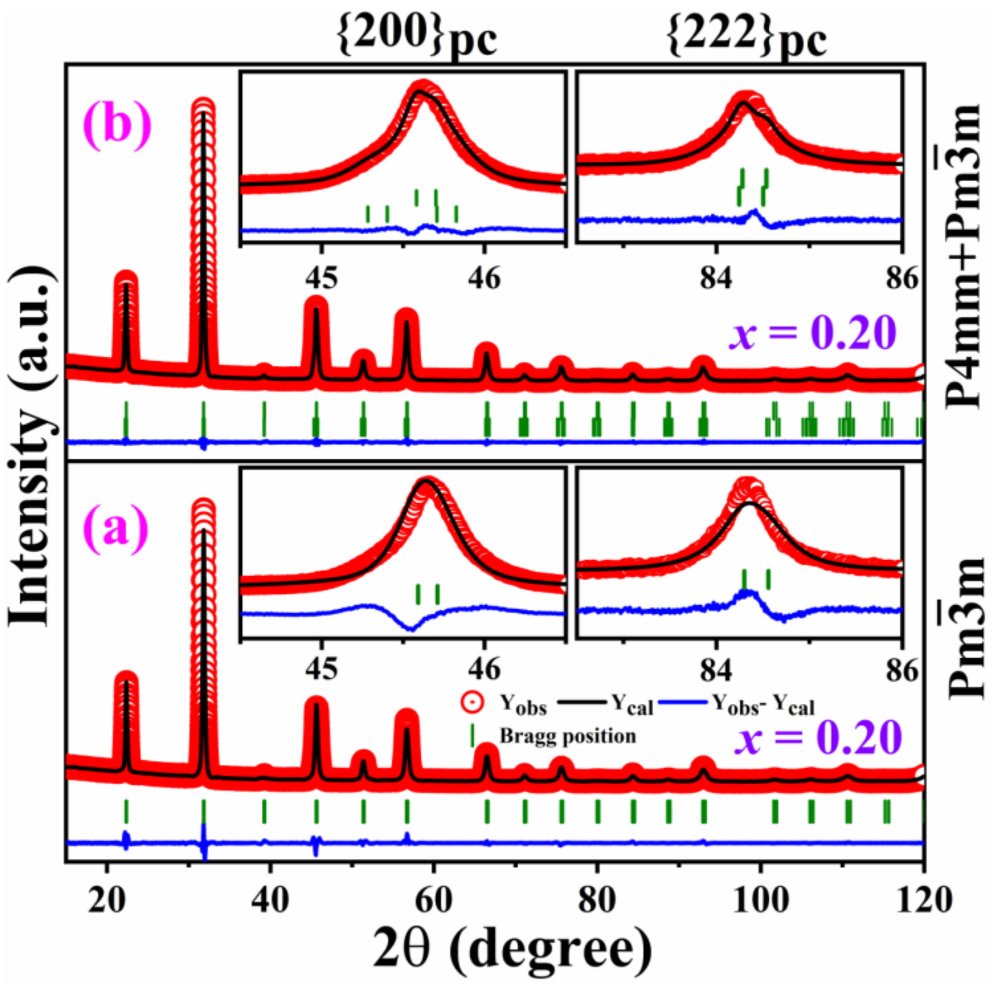

Figure S3. Rietveld fitted x-ray powder diffraction pattern of 0.8KNN-0.2BST using **(a)** $Pm\bar{3}m$, and **(b)** $P4mm + Pm\bar{3}m$ model. The inset shows magnified view of the fitted $\{200\}_{pc}$ and $\{222\}_{pc}$ reflections.

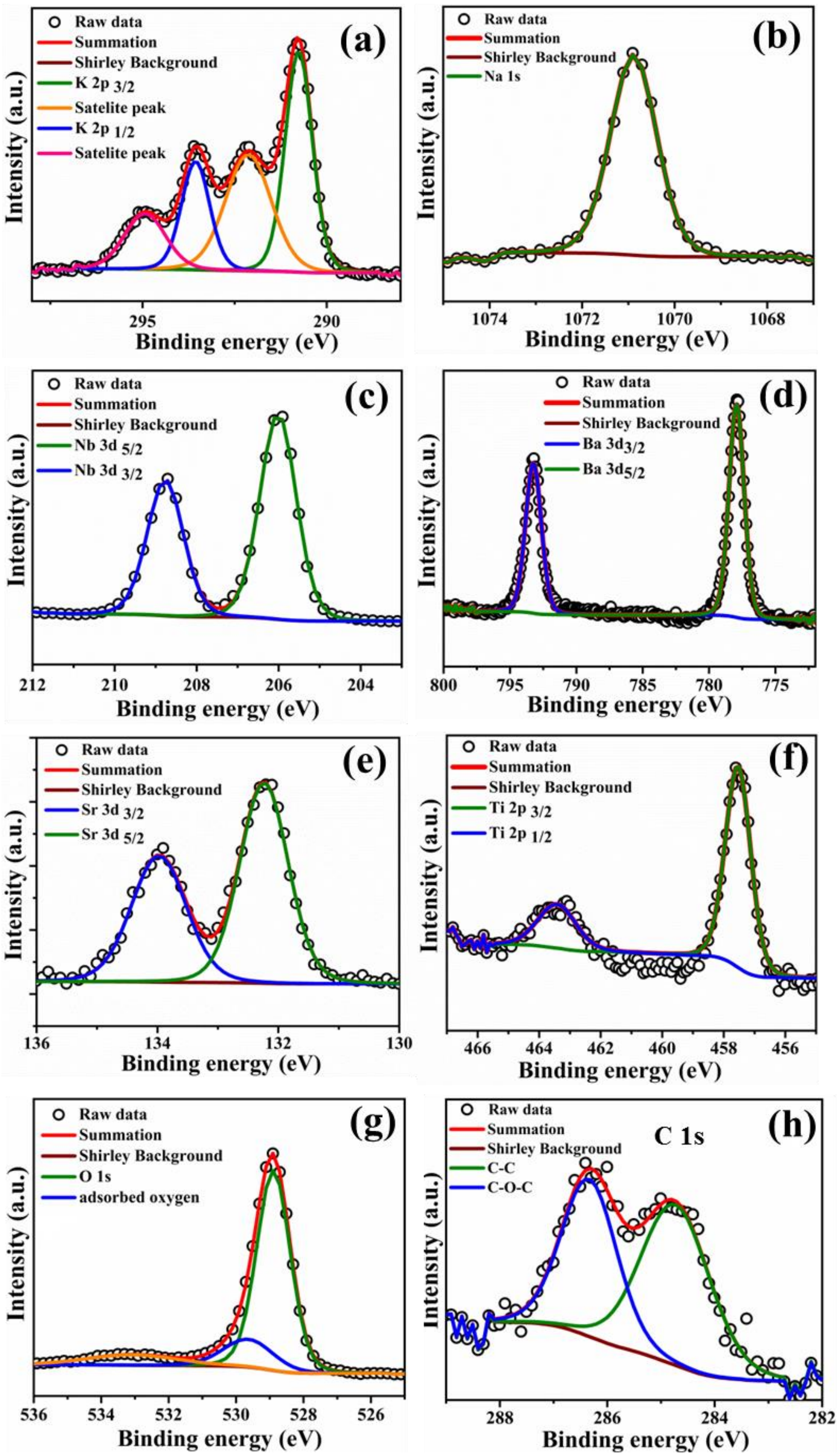

Figure S4. XPS spectra of each element in 0.9KNN-0.1BST solid solution **(a)** K 2p, **(b)** Na 1s, **(c)** Nb 3d, **(d)** Ba 3d, **(e)** Sr 3d, **(f)** Ti 2p, **(g)** O 1s and **(h)** C 1s.

1s signal from the carbon present on surface following the air exposure was used for charge correction. We observe the characteristic peaks for KNN-BST, a K 2p doublet emerges at binding energies 293.56 and 290.78 eV, Na 1s emerge at binding energy 1070.90 eV, Nb 3d doublet emerges at binding energies 208.74 and 205.99 eV, Ba 3d doublet emerges at binding energies 793.21 and 777.90 eV, Sr 3d doublet emerges at binding energies 133.99 and 132.25 eV, Ti 2p doublet emerges at binding energies 463.46 and 457.55 eV, O 1s emerge at binding energy 528.90 eV.

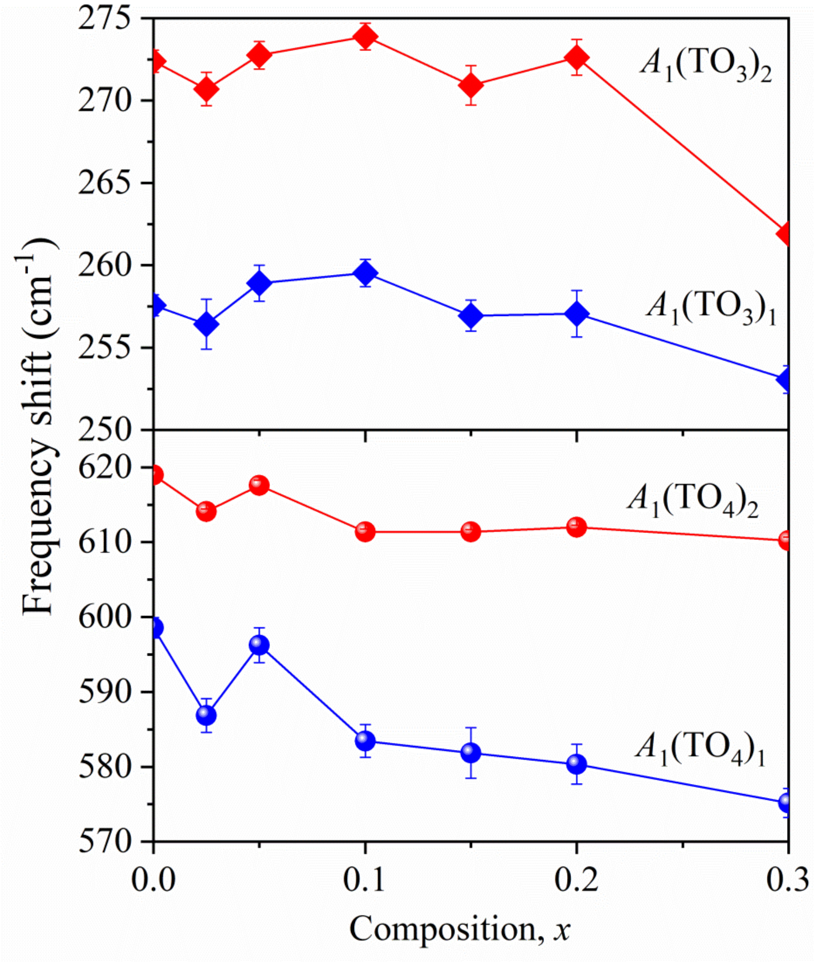

Figure S5. The frequency shift of the $A_1(TO_3)$ and $A_1(TO_4)$ modes as a function of composition.

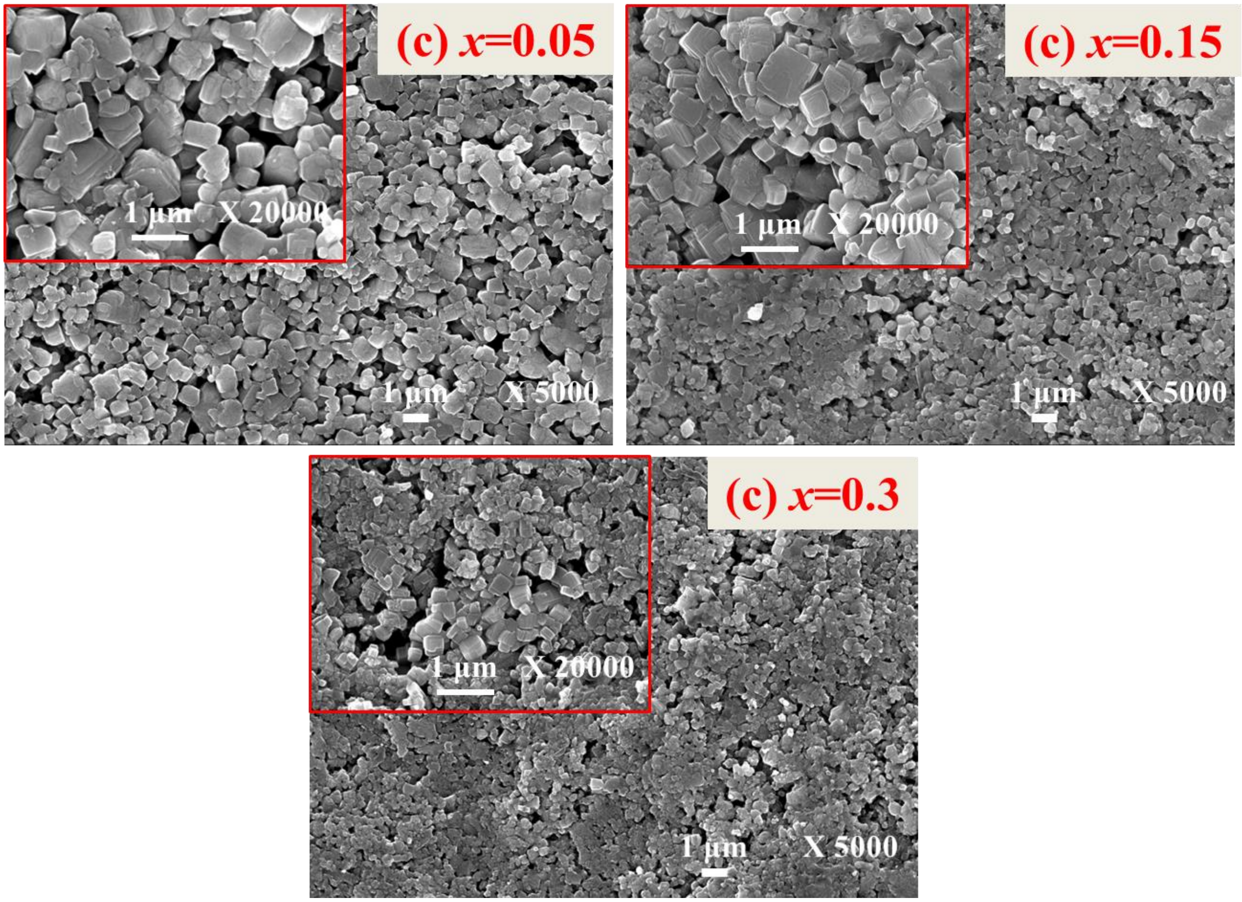

Figure S6. FESEM micrographs of the (1-x)KNN-xBST ceramics for **(a)** x = 0.05, **(b)** x = 0.15, and **(c)** x = 0.30.

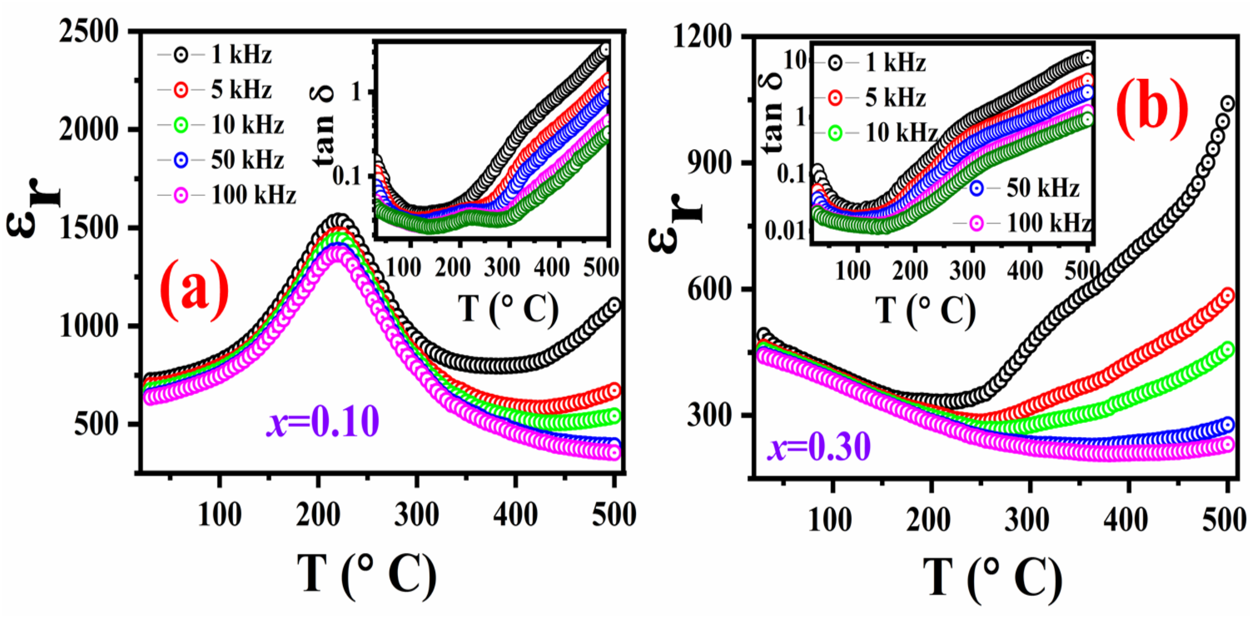

Figure S7. Variation of dielectric constant and dielectric loss as a function of temperature at selected frequencies of (1-x)KNN-xBST ceramics for **(a)** x = 0.10, **(b)** x = 0.30.

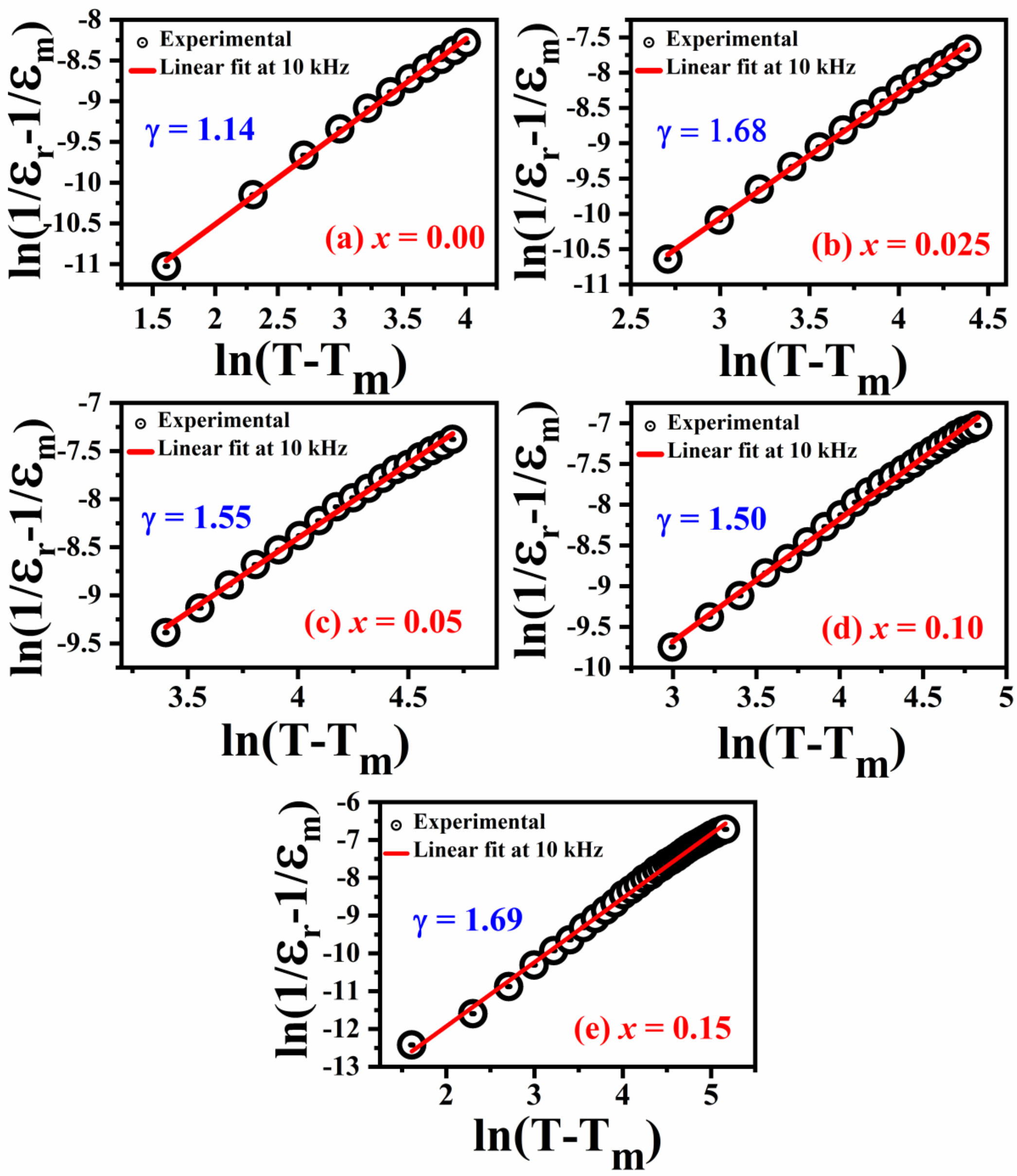

Figure S8. Modified Curie Wiess law plot ($\ln(1/\varepsilon_r - 1/\varepsilon_m)$ versus $\ln(T - T_m)$) of the (1-*x*)KNN-*x*BST ceramics for (a) $x = 0$, (b) $x = 0.025$, (c) $x = 0.05$, (d) $x = 0.1$, and (e) $x = 0.15$

Supplementary Tables:

Table S1. A brief review on the crystal structure and physical properties of KNN based solid solutions reported by various research groups.

Table S2. Refined structural parameters obtained from Rietveld refinement of (1-*x*)KNN-*x*BST ($0 \leq x \leq 0.3$) solid solution.

Table S3. Grain size of (1-*x*)KNN-*x*BST ceramics for $0 \leq x \leq 0.3$.

Table S1. A brief review on the crystal structure and physical properties of KNN based solid solutions reported by various research groups.

Sl. No.	Research group	Details of sample composition	Crystal structure with symmetry	Properties	Ref.
1	Park *et al.* [28]	(1-*x*)($K_{0.5}Na_{0.5}$)NbO_3- *x*$CaTiO_3$	Orthorhombic for $x \leq 0.03$<br><br>Orthorhombic+Tetragonal for 0.03 < x < 0.07<br><br>Tetragonal for $x \geq 0.07$	The MPB composition x = 0.05 shows highest relative density (97%), d_{33} (241 pC/N), K_p(0.41) and $\varepsilon_3^T/\varepsilon_0$(1316).<br><br>With increase in x the ceramic shows relaxor behaviour.	J. Appl. Phys. 102, 124101 (2007)
2	Liang *et al.* [29]	(1-*x*)($K_{0.5}Na_{0.5}$)NbO_3-*x*$Bi_{0.5}K_{0.5}TiO_3$	Orthorhombic for $x < 0.02$<br><br>Orthorhombic and Tetragonal phases at $x = 0.2$<br><br>Tetragonal structure for $x > 0.03$	The ceramics with x=0.02 shows enhanced electrical properties (d_{33} = 251 pC/N, k_p = 0.49, $\varepsilon_3^T/\varepsilon_0$ = 1260, tan δ = 0.03 and T_C = 376 °C) owing to the formation of a MPB and shifting of polymorphic transition temperature ($T_{O\text{-}T}$) near RT.	J. Phys. D: Appl. Phys. 41, 115413 (2008)

3	Liang *et al.* [30]	(1-*x*)$(K_{0.5}Na_{0.5})NbO_3$-$xBiScO_3$		A decrease in the grain size as well as T_C value with increase in BS content has been reported.<br><br>The diffuse nature of phase transition and frequency dispersion of the dielectric constant indicated the relaxor nature of the ceramics.<br><br>The 4 mol% $BiScO_3$ shows broad dielectric maximum with dielectric constant of 2500 from 100 to 300°C, suitable for high temperature capacitors applications.	J. Appl. Phys. 104, 044104 (2008).
4	Guo et al. [31]	(1-*x*)$(K_{0.5}Na_{0.5})NbO_3$-$xLiNbO_3$	Orthorhombic for $x \leq 0.05$<br><br>Orthorhombic+Tetragonal for $0.05 < x < 0.07$<br><br>Tetragonal for $x \geq 0.07$	High Curie temperature (>450 °C) has been reported.<br><br>The ceramics show enhanced piezoelectric and electromechanical responses (d_{33} = 200–235 pC/N, k_p = 38%–44%, and k_t = 44%–48%) in the MPB compositions.	*Appl. Phys. Lett.* **85,** 4121 (2004)
5	Kosec *et al.* [32]	(1-*x*) $K_{0.5}Na_{0.5}NbO_3$-$xSrTiO_3$	Pseudo-cubic distortion for 0.15 to 0.25	Relaxor behavior in the composition range x = 0.15 to 0.25 has been observed.<br><br>x = 0.15 shows highest dielctric permitivity (>3000)	*J. Mater. Res.* **19**, 1849-1853 (2004)
6	Sun *et al.* [33]	(1-*x*)$(K_{0.5}Na_{0.5})NbO_3$-$xBiScO_3$	Orthorhombic phase for x < 0.015<br><br>Orthorhombic + Pseudo-cubic for $0.005 \leq x \leq 0.02$	In this MPB region an enhanced ferroelectric (P_r = 24.4 μC/cm2) and piezoelectric properties (d_{33} = 203 pC/N) are observed.	J. Am. Ceram. Soc. 92, 130-132 (2009)

			Pseudo-cubic phase for $x > 0.02$		
7	Park et al. [34]	$0.95(K_{0.5}Na_{0.5})NbO_3$-$0.05BaTiO_3$		A high piezoelectric constant (d_{33} = 225 pC/N) has been reported.	Appl. Phys. Lett. 89, 062906 (2006)
8	Zuo *et al.* [35]	$(1-x)(K_{0.5}Na_{0.5})NbO_3$-$xBiAlO_3$	Orthorhombic for $x = 0$<br><br>Orthorhombic + Tetragonal for $0.005 \leq x \leq 0.01$<br><br>Pseudo-cubic for $x > 0.02$	The $x = 0.01$ composition shows enhanced ferroelectric (P_r = 23.6 μC/cm^2) and piezoelectric properties (d_{33} = 202 pC/N) owing to the existence of MPB near RT.<br><br>A decrease in average grain size, T_{O-T} and T_C with increase in x has been reported.	J. Alloys Compd. 476, 836-839 (2009)

Table S2. Refined structural parameters obtained from Rietveld refinement of (1-*x*)KNN-*x*BST ($0 \leq x \leq 0.3$) solid solution.

Composition x	Space Group	χ^2	a (Å)	b (Å)	c (Å)	Volume ($Å^3$)	Phase Fraction (%)
0.00	*Amm2*	1.41	3.9420	5.6400	5.6715	126.09	100 (O)
0.025	*Amm2+P4mm*	5.08	3.9523	5.6497	5.6656	126.51	71.75(O)
			3.9733	3.9733	3.9926	63.03	28.25(T)
0.05	*Amm2+P4mm*	5.20	3.9555	5.6415	5.6574	126.24	55.41(O)
			3.9708	3.9708	4.0119	63.26	44.59(T)
0.10	*Amm2+P4mm*	7.12	3.9681	5.6325	5.6467	126.21	45.37(O)
			3.9638	3.9638	4.0095	62.99	54.63(T)
0.15	*Amm2+P4mm*	8.25	3.9603	5.6619	5.6524	126.74	30.34O)
			3.9691	3.9691	3.9934	62.91	69.66(T)
0.20	$P4mm+Pm\bar{3}m$	3.44	3.9676	3.9676	4.0012	62.99	33.71(T)
			3.9769	3.9769	3.9769	62.90	66.29 (C)
0.30	$Pm\bar{3}m$	7.29	3.9768	3.9768	3.9768	62.89	100 (C)

Table S3. Grain size of (1-*x*)KNN-*x*BST ceramics for $0 \leq x \leq 0.3$.

Composition *x*	Grain Size (μm)
0.00	2.52
0.025	0.38
0.05	0.42
0.10	0.29
0.15	0.34
0.20	0.22
0.30	0.17
